# Modeling the Effect of Bovine Viral Diarrhea Virus in Australian Beef Herds

**DOI:** 10.3389/fvets.2021.795575

**Published:** 2021-12-14

**Authors:** Jake Fountain, Marta Hernandez-Jover, Carsten Kirkeby, Tariq Halasa, Jennifer Manyweathers, Yiheyis Maru, Victoria Brookes

**Affiliations:** ^1^Graham Centre for Agricultural Innovation (An Alliance Between Charles Sturt University and NSW Department of Primary Industries), School of Agricultural, Environmental and Veterinary Sciences, Charles Sturt University, Wagga Wagga, NSW, Australia; ^2^School of Agricultural, Environmental and Veterinary Sciences, Charles Sturt University, Wagga Wagga, NSW, Australia; ^3^Section of Animal Welfare and Disease Control, Institute of Veterinary and Animal Sciences, Faculty of Medical and Health Sciences, University of Copenhagen, Frederiksberg, Denmark; ^4^Commonwealth Scientific and Industrial Research Organisation Land and Water, Canberra, ACT, Australia; ^5^Sydney School of Veterinary Science, Faculty of Science, The University of Sydney, Camperdown, NSW, Australia

**Keywords:** BVDV, simulation, model, Australia, beef, production, bovine, viral

## Abstract

Bovine viral diarrhea virus (BVDV) is an economically important disease in Australian beef farming. The disease typically results in low-level production losses that can be difficult to detect for several years. Simulation modeling can be used to support the decision to control BVDV; however, current BVDV simulation models do not adequately reflect the extensive farming environment of Australian beef production. Therefore, the objective of this study was to develop a disease simulation model to explore the impact of BVDV on beef cattle production in south-east Australia. A dynamic, individual-based, stochastic, discrete-time simulation model was created to simulate within-herd transmission of BVDV in a seasonal, self-replacing beef herd. We used the model to simulate the effect of herd size and BVDV introduction time on disease transmission and assessed the short- and long-term impact of BVDV on production outputs that influence the economic performance of beef farms. We found that BVDV can become established in a herd after a single PI introduction in 60% of cases, most frequently associated with the breeding period. The initial impact of BVDV will be more severe in smaller herds, although self-elimination is more likely in small herds than in larger herds, in which there is a 23% chance that the virus can persist for >15 years following a single incursion in a herd with 800 breeders. The number and weight of steers sold was reduced in the presence of BVDV and the results demonstrated that repeat incursions exacerbate long-term production losses, even when annual losses appear marginal. This model reflects the short- and long-term production losses attributed to BVDV in beef herds in southeast Australia and provides a foundation from which the influence and economic utility of BVDV prevention in Australian beef herds can be assessed.

## Introduction

Endemic diseases are responsible for significant economic losses in the Australian beef industry. A report commissioned in 2015 indicated that bovine viral diarrhea virus (BVDV) was the second-most economically significant endemic disease affecting Australian beef production (1). BVDV is a small, highly infectious, enveloped RNA virus of the genus Pestivirus (2). Serological studies have shown that cattle in all states across Australia are exposed to the virus, with a herd-level seroprevalence between 48% (Tasmania) and 86% (South Australia) (3). A review of BVDV in the eastern states of Australia conducted in 2012 revealed that the national herd-level seroprevalence of the disease has remained at ~60% since 1967 (3, 4).

Loss of production in beef herds is typically characterized by the reproductive effects of BVDV. Early embryonic death and abortion as a result of virus exposure can lead to reduced pregnancy rates and increased calving intervals (5). This effect on reproduction can be severe if a naïve cattle population is exposed to the virus, especially during the breeding period. In-utero infection from 30 to 120 days gestation is likely to result in the birth of a persistently infected (PI) calf (2, 6). PI calves are the main source of viral persistence within a cattle population and the presence of a single PI calf has been attributed to herd seroconversion rates of up to 97% in a six-month period (7–10). PI calves are also attributed with increased calf mortality, with a 35–50% probability of death or culling before one year of age (10). In contrast, transiently infected (TI) animals (susceptible animals that are acutely infected) only shed the virus for up to 14 days and are generally subclinical, making them an inefficient source of horizontal transmission between herds (6, 8).

In a herd with endemic BVDV infection, the reproductive and immunosuppressive effects of the disease typically result in low-level annual production losses which can be difficult to detect, but the loss over several years can be significant (11). The production losses attributed to BVDV can influence the export market. Australia is the world's third largest exporter of beef products and is able to compete for global markets due to superior disease status and production of high-quality products (12). As an OIE-Listed disease, BVDV influences international trade under the Sanitary and Phytosanitary Agreement (13, 14). Strategies for the control of BVDV have become increasingly important, with some countries using test and cull schemes and others using a combined approach with vaccination (2, 15).

Despite these interventions, the success of nationwide control of BVDV is highly variable and modeling has been used to study country-specific disease dynamics and facilitate decisions for disease control (16). Disease modeling can quantify the effect of specific disease control measures on morbidity, animal health and welfare, as well as economic impacts, effects on reproduction and subclinical disease (17). Simulation modeling of BVDV has been used by several countries to justify the implementation of nationwide control and disease mitigation strategies (16, 18, 19). However, dairy systems predominate the production industries in most of these countries. Due to the role of PI calves in BVDV transmission, differences in reproductive and calf-rearing practices between dairy and beef production systems mean that BVDV simulation models for the dairy industry do not reflect the effect of the disease in beef systems (9).

Simulation models have been used to assess the effect of BVDV in beef herds in Scotland, Ireland, United States and France (20–24). These studies used modeling to assess the economic utility of BVDV control strategies, as well as the benefits of on-farm mitigation strategies (17). In the absence of an official national BVDV control program, the responsibility of BVDV control in Australia falls on individual producers. Therefore, the Australian beef industry would benefit from a BVDV simulation model to guide mitigation of the disease at farm-level. Existing BVDV models simulate dairy production systems or beef systems in which cattle are housed in close quarters for a portion of the year, which is not typical of beef production in Australia. Han et al. (25) illustrated a difference in the impact of BVDV on pastoral beef farms when compared to other modeling studies, likely due to differences in housing and farm management. These findings demonstrate the need for country specific BVDV simulation modeling that is able to reflect the management practices of different beef farms.

The objective of this study was to develop a disease simulation model to investigate the impact of BVDV on Australian beef farms and inform the need for intervention in environments typical of beef farming in southeast Australia. The study compares the short- and long-term impacts of BVDV on beef production to highlight the production losses that can occur in the absence of disease prevention strategies.

## Materials and Methods

### Simulation Model

A dynamic, individual-based, stochastic discrete-time simulation model was created using the software R (26) to assess the effect of BVDV on a beef herd representative of seasonal, single-calving, self-replacing beef production systems in south-eastern Australia. The annual production calendar is based on the “Beef Calendar of Operations” published by the local government for south-east NSW (27). Simulations in which BVDV transmission was modeled (Scenario 1–3) were used to assess the short- and long-term effect of BVDV on beef production parameters, compared to a baseline scenario without BVDV (Scenario 0). Mitigation strategies were not simulated. The total number of infected animals was an additional output of interest for Scenario 1–3. BVDV transmission was characterized according to herd size and timing of virus introduction, which also allowed for comparison of simulation output parameters to published values for external validation of the model.

On each day of the simulation (one time-step), the model was updated in five stages: (1) disease transmission; (2) movements between groups on the farm; (3) removal of animals (through deaths, culls or sales); (4) pregnancy status; and (5) addition of animals (through purchases or births). Model parameters including herd structure and contact rates are described below. A Bernoulli process was used to model stochasticity for the probability of daily infection, mortality risk, probability of conception and daily probability of abortions. Sensitivity analysis was conducted to assess the influence of model parameters on the outputs of interest, as described below.

### Herd Structure

The herd consisted of bulls (B), cull cows (CC), weaned steers (WS), weaned heifers (WH), first-calving cows (FC) and mature cows (MC). Each animal group was assigned to a single virtual paddock (Paddock 1–5, respectively) with the exception of MCs, which were split into age-related groups (≥ Paddock 6). The maximum number of MC paddocks was based on the bull-to-cow ratio and varied according to breeding herd size (minimum two MC paddocks and maximum 32 MC paddocks for a breeding herd of 50 and 800 animals, respectively).

**Figure 1:**
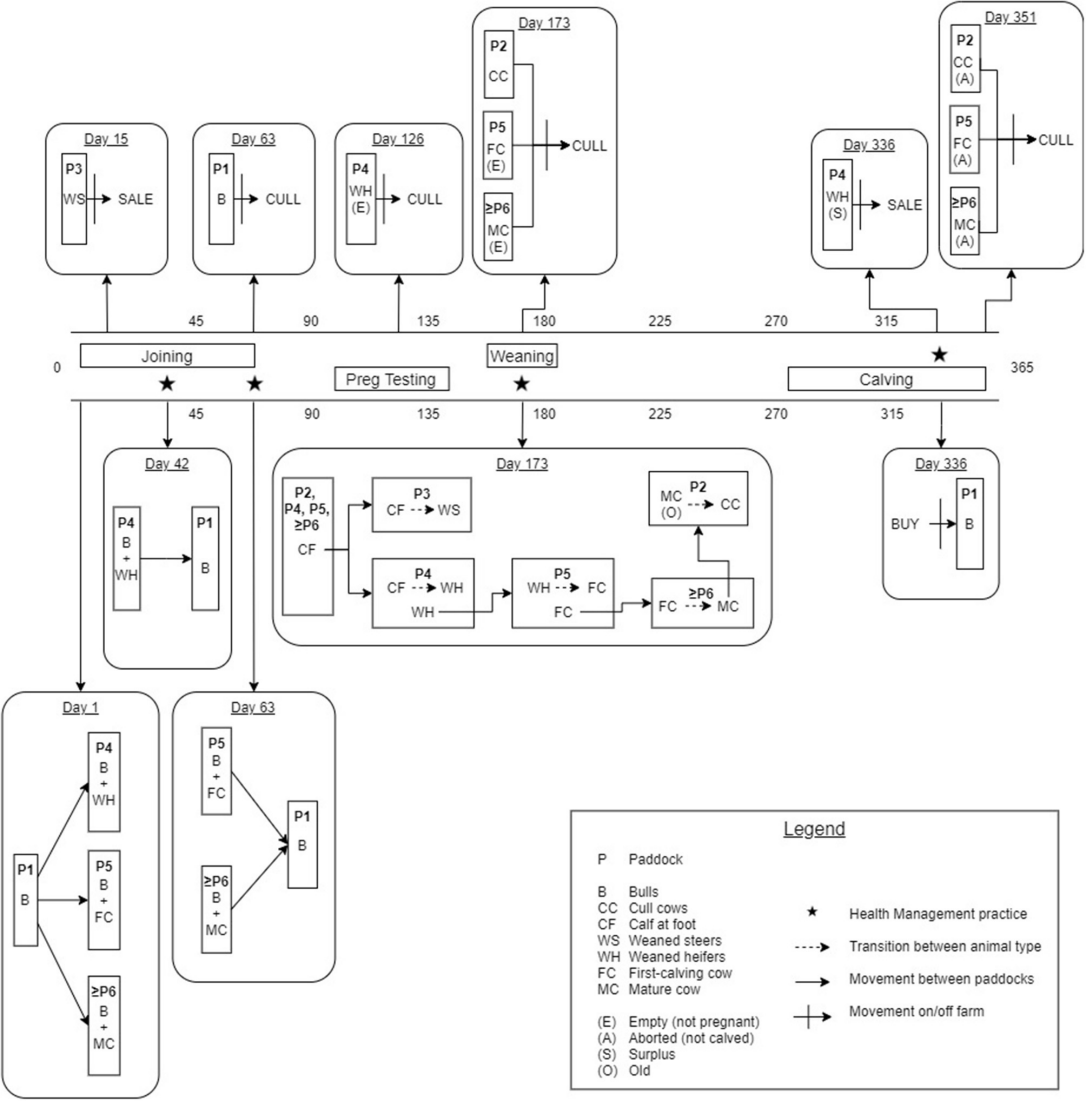
Schematic of the annual production calendar, animal movements and transitions between animal groups for the simulated beef herd.

Figure 1 provides a detailed illustration of the annual production calendar and animal movements used in the simulation model. Bulls were distributed between the WH, FC and MC paddocks (≥ Paddock 4) to simulate the start of the breeding period (joining). An estrus counter for each individual breeding animal (WH, FC and MC) was decreased by one unit daily to simulate the duration of the estrus cycle, and was predetermined from a uniform distribution of 18–24 days for each individual animal (reproduction parameters are described in Table 1). When the counter reached zero, the animal had a chance to conceive (see Table 1). The probability of conception for FC and MCs was dependent on their estrus cycle number and BVDV infection status (Tables 1, 2). WHs had the same probability of conception for all estrus cycles because they did not undergo uterine involution prior to joining. If conception did not occur, the estrus counter was restarted. If the animal conceived, a gestation period was assigned from a PERT distribution of range 279–287 (mode 283). At the start of every joining period, a fixed percentage of cows and heifers were made infertile, to reflect the expected infertility rate of heifers and cows (Table 1). To simulate seasonal breeding, bulls were removed from the WH and FC/MC paddocks at days 42 and 63 calendar year, respectively, based on industry recommendations (45, 46). Bulls were randomly selected and culled annually after the breeding period, according to the annual bull cull rate and an average life expectancy of 3 years (Table 1).

**Table 1:** Parameters used to represent a self-replacing beef system in south-east Australia in a BVDV simulation model.

**Parameter**	**Value**		**References**
Breeding herd size		50, 100, 200, 400, 800	(28)
Heifer retention		0.20	
Bull: cow ratio		1:20	
Maximum cow age (years)		10	(29)
Bull cull rate (per year)		0.33	(30)
Age of first joining (days)		450	(31)
Oestrus interval (days)		Uniform (18, 24)	(32)
Uterine involution (days)		PERT (36, 45, 55)	(32, 33)
Gestation period (days)		PERT (279, 283, 287)	(34)
Annual probability of infertility			
	Heifers	0.02	(20)
	Cows	0.08	
Conception rate			
	Heifers	0.70	(35)
	Cows (1st oestrus)	0.55	
	Cows (2nd oestrus)	0.67	
	Cows (3rd oestrus)	0.70	
Natural abortion rate (annual)		0.035	(33)
Calf sex (male: female)		0.50	(20)
Calving target		0.90	(28)

**Table 2:** Disease parameters used in a simulation model of BVDV in a self-replacing beef herd.

**Parameter**	**Value**		**References**
Probability of effective contact	PI within-herd (*p*_PI_)	$\frac{PERT\;\left(0.03,\;0.11,\;0.50\right)}{N_{j}}$	(33)
	PI between-herd (*p*_PIk_)	*p*_*PI*_ × *PERT* (0.1, 0.2, 0.4)	(20, 36, 37)
	TI within-herd (*p*_TI_)	*p*_*PI*_ ×0.05	(33)
	TI between-herd	0	(7)
Latency period (days)		4	(36)
Shedding period (days)		PERT (7, 10, 14)	(6, 36, 37)
Maternal antibody duration (days)		PERT (120,180, 240)	(36)
Conception rate (BVDV infected)	Heifers	0.42	(35)
	Cows (1st oestrus)	0.33	
	Cows (2nd oestrus)	0.39	
	Cows (3rd oestrus)	0.42	
TI reduced fertility duration (days)		U (42, 60)	(38, 39)
Morbidity risk	TI adult	$1-e^{-\left(U\frac{\left(0.10,\;0.3\right)}{365}\right)}$	(40)
	TI calf	$1-e^{-\left(PERT\frac{\left(0.02,\;0.24,\;0.69\right)}{365}\right)}$	(41)
Annual mortality rate	Adult (*m_*a*_*)	PERT (0.008, 0.017, 0.024)	(28)
	Healthy calf (*m_*c*_*)	PERT (0.03, 0.045, 0.06)	(33, 42, 43)
	Morbid calf (*m_*m*_*)	PERT (0.35, 0.5, 0.66)	(10, 44)

*N_j_, Number of animals in group j*.

Any WH that was not pregnant at day 126 following the end of the breeding period (simulated pregnancy testing) was culled from the herd (Figure 1). FC and MCs with a calf at foot (CF) that were empty at pregnancy testing were culled at weaning (27). CFs remained in the same group as their dam until weaning at day 173 of the calendar year (6–9 months of age). At weaning, all CFs were distributed into Paddocks 3 and 4, where they became WS and WH, respectively. The WH bred in this season were moved to Paddock 5 to become the new FCs and the previous FCs were distributed to the MC paddock with the fewest animals. If the breeding herd size was sufficient to meet targets in the next joining period, the oldest MCs were moved to Paddock 2 and became CCs, to be culled the following year at weaning (29).

A CF was added to the herd when the gestation counter was equal to the breeding animal's assigned gestation period, signifying the end of gestation. In the absence of BVDV, a BVDV-susceptible, healthy calf was produced with a 0.5 probability of being male (20). The estrus counter for the breeding animal was recommenced from between 36 and 55 days to represent uterine involution, with the exception of FCs which were assigned an additional 20–30 days of involution (33, 34). Any breeding animal that did not produce a calf (due to abortion following pregnancy testing) was culled after the calving period. One month prior to joining, bulls were purchased to maintain a bull-to-cow ratio of 1:20 (30). WH in Paddock 4 were selected for retention based on live weight on day 336 of the calendar year, prior to the joining period. The lightest WHs were removed from the herd as sale animals if they were not required to maintain breeding herd numbers (Figure 1). If numbers of replacement heifers were not sufficient to maintain breeding herd targets, the youngest CCs from Paddock 2 were moved back into the MC paddocks for joining. All WS were removed from the herd as sale animals at Day 15 of the calendar year at 14–16 months of age. WS that were 380–500 kg satisfied the weight requirements for a premium price at sale (47).

### Disease Dynamics

Infection dynamics for BVDV were based on an extension of the Reed-Frost model for individual-based, discrete-time disease modeling (48). Disease transmission on any day was dependent on the total number of effective animal contacts and the total number of TI and PI animals present within the population. Infection from a PI animal to a susceptible individual could occur through direct contact between cattle within a group, or *via* aerosols, vectors or fomites between groups (8, 9). Previous studies have determined that spread from TI cattle to other groups is negligible and therefore, transmission of the virus due to contact with TI animals could only occur within a group (7, 49).

Indirect transmission of BVDV has been demonstrated *via* shared needles during vaccination and reused rectal gloves during pregnancy testing (8) and therefore, increased probability of infection during health management was reflected in the simulation model through vaccination and pregnancy testing. Four vaccinations were simulated annually (Figure 1): initial CF vaccination (Day 42), second CF vaccination (Day 63), booster CF vaccination at weaning and booster B, WH, FC and MC vaccination prior to joining (Day 336).

When accounting for all routes of transmission of BVDV, the daily risk of infection for an individual animal in group *j* (λ_*j*_) is described by equation 1:


(1) $$\begin{array}{c} \lambda_{j}=1-({\left(1-p_{PI}\right)}^{I_{PIj}}\times {\left(1-p_{TI}\right)}^{I_{TIj}}\times \Pi_{k;k\neq j}{\left(1-p_{PIk}\right)}^{I_{PIk}}\\ \times {\left(1-p_{PI}\right)}^{I_{PIh}}{}^{*}\times {\left(1-p_{TI}\right)}^{I_{TIh}}{}^{*}) \end{array}$$

^*^For animals involved in management practices on that day.

in which: *I*_PIj_ = the number of persistently infected animals within paddock *j*; *p*_PI_ = the probability of daily effective contact between individuals for PI transmission within paddock *j*; *I*_TIj_ = the number of transiently infected animals within paddock *j*; *p*_TI_ = the probability of daily effective contact between individuals for TI transmission within paddock *j*; *I*_PIk_= the number of persistently infected animals in paddock *k*; *p*_PIk_ = the probability of daily effective contact from PI animals in paddock *k* to animals in paddock *j* (*k* = 1, …, *n, k* ≠ *j*). *I*_PIh_ and *I*_TIh_ represent the number of PI animals and the number of TI animals involved in a health management practice, respectively. The value for *p*_PI_ in the current model study was derived from values used in Han, Weston (33), and the values for *p*_PIk_ and *p*_TI_ were scaled according to *p*_PI_ (Table 2).

### Morbidity, Mortality and BVDV Effect on Pregnancy

BVDV infection in TI females can cause oophoritis which will reduce the probability of conception (38, 39). In the model, non-pregnant animals that recovered from transient infection had a reduced probability of conception (the same probability of conception as an animal with active BVDV infection) for 42–60 days (Table 2). If an animal became infected while pregnant, the likelihood of abortion, development of a PI calf, weak-born calf and the probability of a calf born with permanent immunity to BVDV was based on McCormick 2010 (Supplementary Table 1). Breeding animals that recovered from transient infection prior to conception produced naïve calves that received passive immunity from colostrum. The duration of maternal immunity was 120–240 days, following which the calf became fully susceptible (36). A pregnant PI animal that did not abort their conceptus always produced a PI calf (6).

The daily individual mortality risk (*m*_*r*_) was derived from the relationship between risks and rates as described by Vynnycky and White (48):


(2) $$m_{r}=1-e^{\left(-\frac{m_{i}}{365}\right)}$$

in which: *e* = Euler's number and *m*_*i*_ = the annual mortality rate based on the animal type (*m*_*a*_, *m*_*c*_ and *m*_*m*_ for adults, healthy calves and morbid animals, respectively). The mortality rates of adult cattle and healthy calves were derived from industry data from Meat & Livestock Australia (28) (Table 2). Most studies suggest that approximately half of all PI calves die within the first year (50, 51). Houe et al. (10) and McCormick et al. (44) found that the annual mortality rate of PI calves was ~35 and 66%, respectively. Therefore, annual PI calf mortality was based on a PERT distribution with 50% as the mode (range 0.35–0.66). Weak-born calves will exhibit high mortality in the first week of life, as well as poor growth rates up to weaning (6, 52, 53). Therefore, in the current model, weak-born calves had the same mortality rate as a PI animal until they reached seven days old. The mortality risk of a TI individual was not affected by BVDV infection.

Immunosuppression due to BVDV infection can increase the risk of secondary infections, causing calf diarrhea and bovine respiratory disease (BRD) (40, 54, 55). However, due to the variations in management practices and BVDV incidence between farms, it is difficult to accurately model the effect of BVDV on the incidence of specific secondary infections, or the effect of secondary infection on mortality. In the current model, risk of secondary infection due to immunosuppression from BVDV was characterized as a reduction in growth rate due to morbidity. All weak-born calves and all but 10% of PI calves were morbid throughout their entire lives [Taylor and Rodwell (52) reported 3 out of 30 PI animals with similar growth performance to healthy cattle]. TI calves < six months old that were infected with BVDV after birth had a risk of becoming morbid for the duration of their infectious period (Table 2). Transient infection is subclinical in 70–90% of adult cattle (40). Therefore, TI adults had a uniform probability of 0.1–0.3 of becoming morbid throughout the duration of their infectious period.

### Modeling Daily Liveweight Gain

The model simulated live weight gain for CF, WS, WH and FC. Daily weight gain was calculated as an animal-specific percentage of individual animal live weight, derived from industry standard targets (Supplementary Table 2). Calves were assigned a birthweight from 32 to 40 kg based on the gestation period of their dam, with longer gestation periods producing heavier calves at birth (46, 56, 57). The growth rate of calves reared by an FC is 10–15% less than that of calves reared by an MC (58). Furthermore, female calves will grow about 5% slower than their male siblings until 400 days of age (59). These variations in weight gain were reflected in the model using animal-specific growth percentages for CF steers, heifers, and FC-reared calves (Supplementary Table 3).

An outbreak of BVDV on a beef herd in central Queensland Australia recorded significant variability in the weight of PI grower steers at weaning and sale (52). There is also evidence that TI animals and calves born weak following intra-uterine infection exhibit sub-optimal growth rates compared to virus negative animals (40, 54, 60). In the current model, weak-born and morbid PI calves had reduced daily live weight gains until 450 days of age (Supplementary Table 2) (52, 61).

### Disease Simulation Scenarios and Model Outputs

Simulations commenced at the start of joining and prior to each scenario (Table 3) the model was run for a five-year burn-in period to reach a stable herd population. Initially, beef production was simulated for a period of 15 years without BVDV to obtain baseline production outputs for an uninfected herd (Scenario 0).

**Table 3:** Scenario and introduction time categories for within-herd simulation of BVDV in an Australian beef herd.

**Scenario**	**Description**
Scenario 0	No disease introduction
Scenario 1	Introduction of a single PI animal every 15 years
Scenario 2	Introduction of a single PI animal every 6 years
Scenario 3	Introduction of a single PI animal every 3 years
**Introduction time**	
1	Day 1–73
2	Day 74–146
3	Day 147–219
4	Day 220–292
5	Day 293–365

In Scenario 1, a single animal (<3 years old) was randomly selected and converted to a PI animal to simulate introduction of a PI animal into the farm from an external source. The model was run for a simulation period of 15 years with no further introduction of the virus. The day of BVDV introduction was recorded and categorized according to the time of year (Table 3). Scenario 1 was used to examine the impact of herd size and introduction time on the infection dynamics of BVDV.

Scenario 2 and 3 examined the impact of BVDV introduction frequency by simulating the introduction of a PI animal at regular intervals over a 15-year period (Table 3). As with Scenario 1, a random animal from the herd was converted to a PI animal in the first year of the simulation, and then again at 6- and 3-year intervals for Scenario 2 and 3, respectively. Each subsequent disease introduction event in Scenario 2 and 3 occurred on the same day of the calendar year to aid internal validation and interpretation of results.

Model outputs of interest were those that contribute to the economic performance of an Australian beef herd, including: the number of heifers retained for breeding, the number of cows culled, the number of bulls purchased, the annual number of steers sold and the number of those steers that were under the target weight to obtain a premium price at sale (47). It is assumed that the number of surplus heifers sold would follow the same trend as steer sales (due to indiscriminate calf deaths) and so, heifer retention was used as a surrogate of heifer sales. An increase in heifer retention will not only imply a reduction in the number of surplus heifers sold but can also be an indicator of poor reproductive performance in the breeding herd when mature cows are sold due to poor conception rates (46).

PI animals are the main contributors to BVDV transmission and persistence within a herd. Therefore, if a PI animal was born following disease introduction, we characterized this as establishment of infection within the herd. Self-elimination of the virus was classified as the last year that any infected animals (TI and PI animals) were present in the herd. The cumulative number of infected (TI and PI) animals was also of interest in those scenarios with BVDV transmission. Simulations were repeated for all scenarios until the model outputs reached convergence, with input variables changing every iteration according to the distributions in Tables 1, 2.

The effect of disease introduction frequency on performance was also demonstrated by taking the “per breeding animal” difference in outputs from Scenarios 1–3 compared to Scenario 0, at the cumulative 5-, 10- and 15-year values for all outputs of interest to enable direct comparisons between herd sizes.

### Model Validation

Internal validation of the model was achieved using the rationalism and the tracing methods (36). Implementation without disease introduction ensured that the breeding herd size and replacement practices were performing as expected, given industry-derived parameters, and to determine the burn-in period of the model. Implementation with BVDV transmission tested variation in outputs in response to variation in transmission parameters. Individual animals were followed throughout the simulations to identify inconsistencies in model activity. The disease status of each newborn calf was recorded annually to ensure that vertical transmission aligned with the disease status of the herd.

External validation was achieved by comparing the model outputs to published data from the BVDV literature. Transmission characteristics of the virus and model outputs such as pregnancy risk, abortion rate and calf deaths were validated against corresponding values from Australian BVDV outbreaks using the modeling scenario, breeding herd size and introduction time that best fit the context of published cases.

### Convergence Testing

The number of iterations required to achieve convergence for each modeling scenario was identified using the method described in Brookes et al. (62). Briefly, the coefficient of variation (CV; standard deviation/mean) of a sample from each model output was calculated. This was repeated, increasing the sample size each time, causing CV to approach zero until the number of iterations (sample size) was sufficient to achieve output stability (CV <0.025; the lower part of a 95% confidence interval). Convergence testing was performed on the 15-year cumulative values (expressed as a proportion of breeding herd size) of the main model outputs of interest for each scenario.

### Sensitivity Analysis

Sensitivity analysis was used to determine the influence of input variables on the outputs of the scenario with the most variation (as determined by convergence testing). Only input variables that changed with every iteration of the model (based on a uniform or PERT distribution) were included in the analysis, as well as breeding herd size and introduction time (Supplementary Table 4). Collinearity was expected between the parameter for “number of effective PI contacts within a paddock” and “number of effective PI contacts between a paddock” and therefore, the latter was omitted from the analysis. The 15-year cumulative values for the number of infected animals, as well as the outputs contributing to economic performance in beef farming, were used in the sensitivity analysis.

Scatter plots were used to assess for collinearity between input parameters. Generalized additive mixed models [GAM model; gamm function in the mcgv package (63)] with a negative binomial link function were used to model the relationships between input and output data with a smooth function to account for non-linear relationships between some input parameters and model outputs. The GAM model then was used to predict the minimum and maximum effect of each independent variable on the model outputs (while all other independent variables were fixed at their median value), which were checked visually to ensure that the GAM model results were sensible.

## Results

### Convergence

The outcome of convergence testing for all scenarios is illustrated in Supplementary Figures 1–4. The output with the most variation over 15 years was the total number of infected animals, followed by the number of bulls purchased. It was deemed that variation in all outputs of interest for all Scenarios was <2.5% after 5,000 iterations.

### Description of BVDV Transmission Characteristics

**Figure 2:**
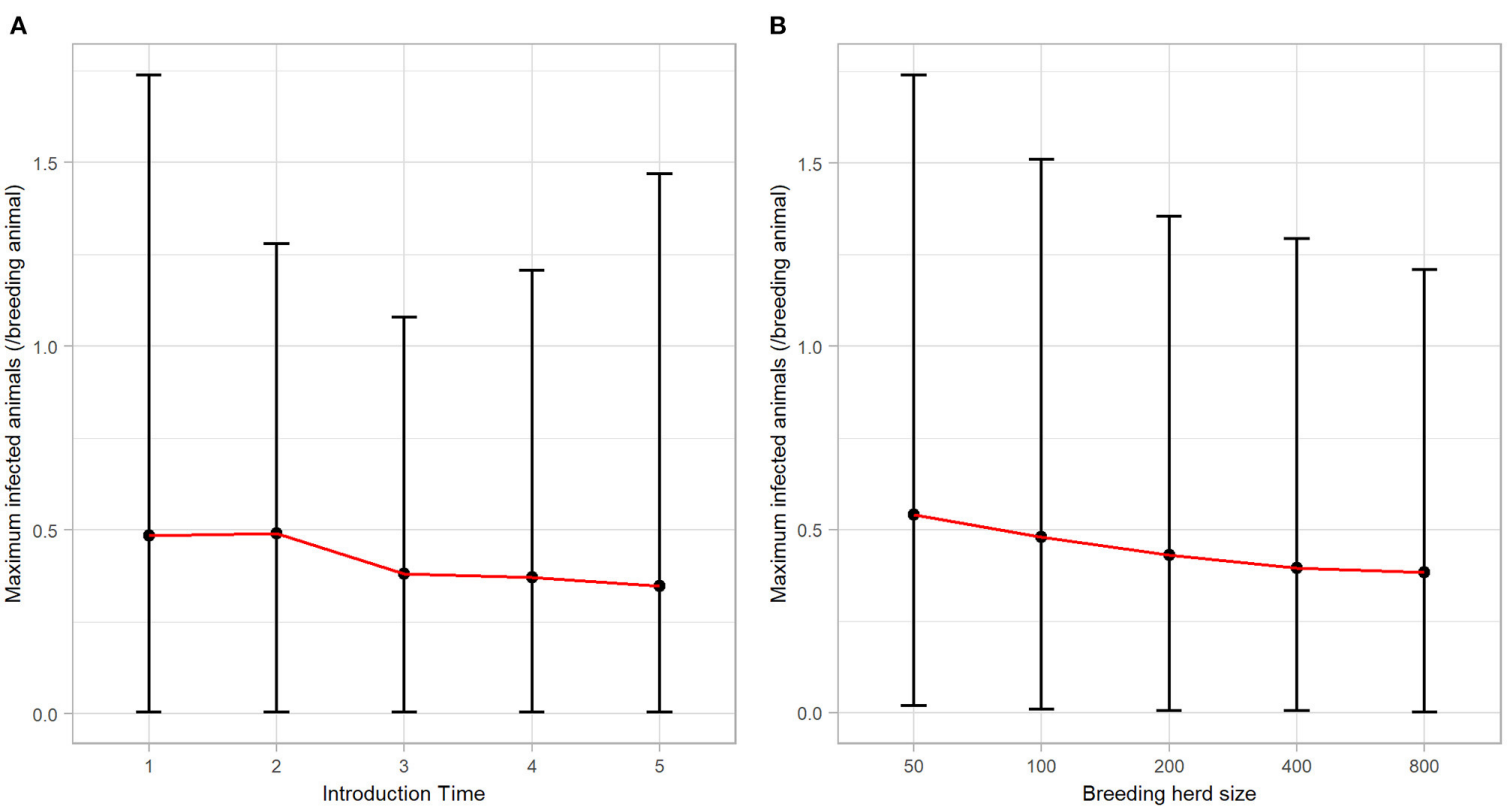
The maximum annual BVDV infections following a single PI introduction in a simulated beef herd. Line plots illustrating the influence of **(A)** introduction time and **(B)** breeding herd size on the number of animals (per breeding animal) that became infected by BVDV in the highest year of BVDV transmission. Points represent the median value, while error bars represent the 95% range.

BVDV became established in 59.66% (*n* = 2983) of all iterations following a single PI introduction and therefore, quantitative analysis of all results that follow are based on these iterations. BVDV was least likely to become established in a herd with 50 breeders and after an incursion at Introduction Time 3 (Table 4). The maximum number of viremic animals following establishment of BVDV was highest (at the median value) after an incursion at Introduction Time 2 (median 0.49 infected animals/breeding animal) and was lowest at Introduction Time 5 (median 0.35 infected animals/breeding animal; Figure 2A). The maximum annual number of viremic animals/breeding animal following a single PI introduction had an inverse relationship to breeding herd size, with 50-breeders resulting in a median 0.54 (95% range 0.02–1.74) infected animals/breeding animal compared to median 0.38 (95% range 0.00–1.21) for 800-breeders (Figure 2B).

**Table 4:** Characteristics of BVDV transmission for a single PI introduction (Scenario 1) in a simulated beef herd.

**Input variable**		**Iterations with infection established (%)**	Iterations^*^ with virus self-elimination (%)
Overall		2,983 (59.66)	2,739 (91.82)
Breeding herd size	50	546 (55.70)	546 (100.0)
	100	563 (56.40)	560 (99.50)
	200	606 (59.70)	585 (96.50)
	400	611 (60.00)	539 (88.20)
	800	657 (66.40)	509 (77.40)
Introduction time	1	911 (91.60)	838 (92.00)
	2	752 (73.90)	687 (91.40)
	3	318 (31.60)	293 (92.00)
	4	421 (41.40)	388 (92.20)
	5	581 (59.90)	533 (91.70)

* *Only iterations in which infection was established are included*.

**Figure 3:**
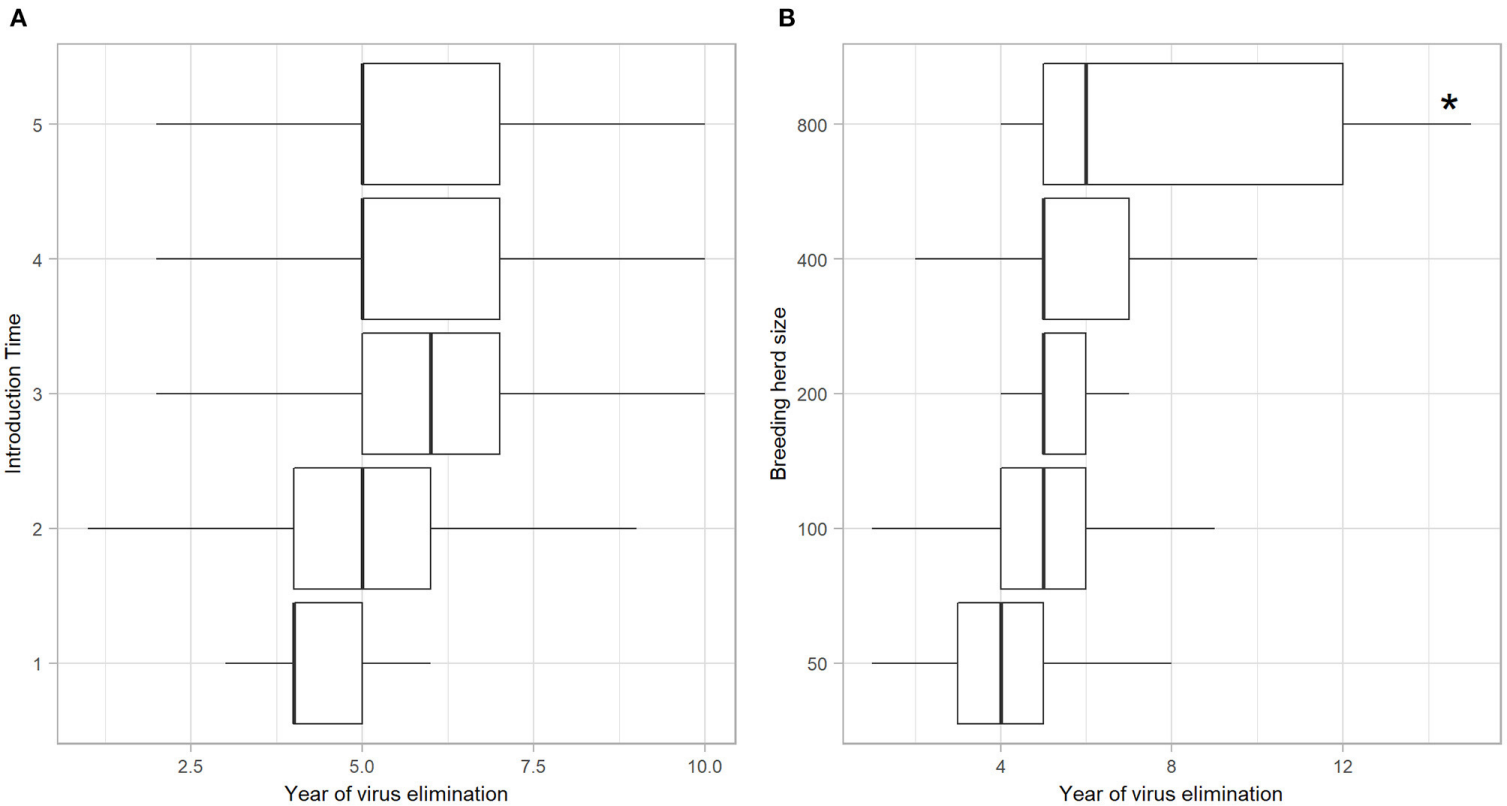
Year of BVDV elimination in those iterations with virus establishment following a single PI introduction in a simulated beef herd. Boxplots illustrating the year at which BVDV self-eliminated from the herd following virus introduction at different times of the production calendar **(A)** and in different herd sizes **(B)**. Boxes represent the 25 and 75% percentiles while whiskers represent the 2.5 and 97.5% percentiles. Outliers removed for improved scale and readability. Asterisk (^*^) indicates that no self-elimination occurred in the 95% range.

Of the iterations that resulted in establishment of BVDV, self-elimination occurred in 91.82% (*n* = 2739) of cases (Table 4). There was no influence of Introduction Time on the likelihood of self-elimination once the disease was established (92% of iterations resulted in self-elimination for all Introduction Times). Introduction Time 1 had the shortest time to self-elimination (median 4 years, 95% range 3–5) and Introduction Time 3 resulted in the longest time to self-elimination (median 6 years, 95% range 2–10; Figure 3A). The virus was most likely to persist beyond the simulation period (15 years) in a herd with 800-breeders and self-eliminated in 100% of iterations with 50-breeders (Table 4). The virus self-eliminated from a herd of 50- and 800-breeders at median 4 years (95% range 1–8) and median 6 years (4–15; ^*^No self-elimination occurred in the upper 95% percentile), respectively (Figure 3B).

**Figure 4:**
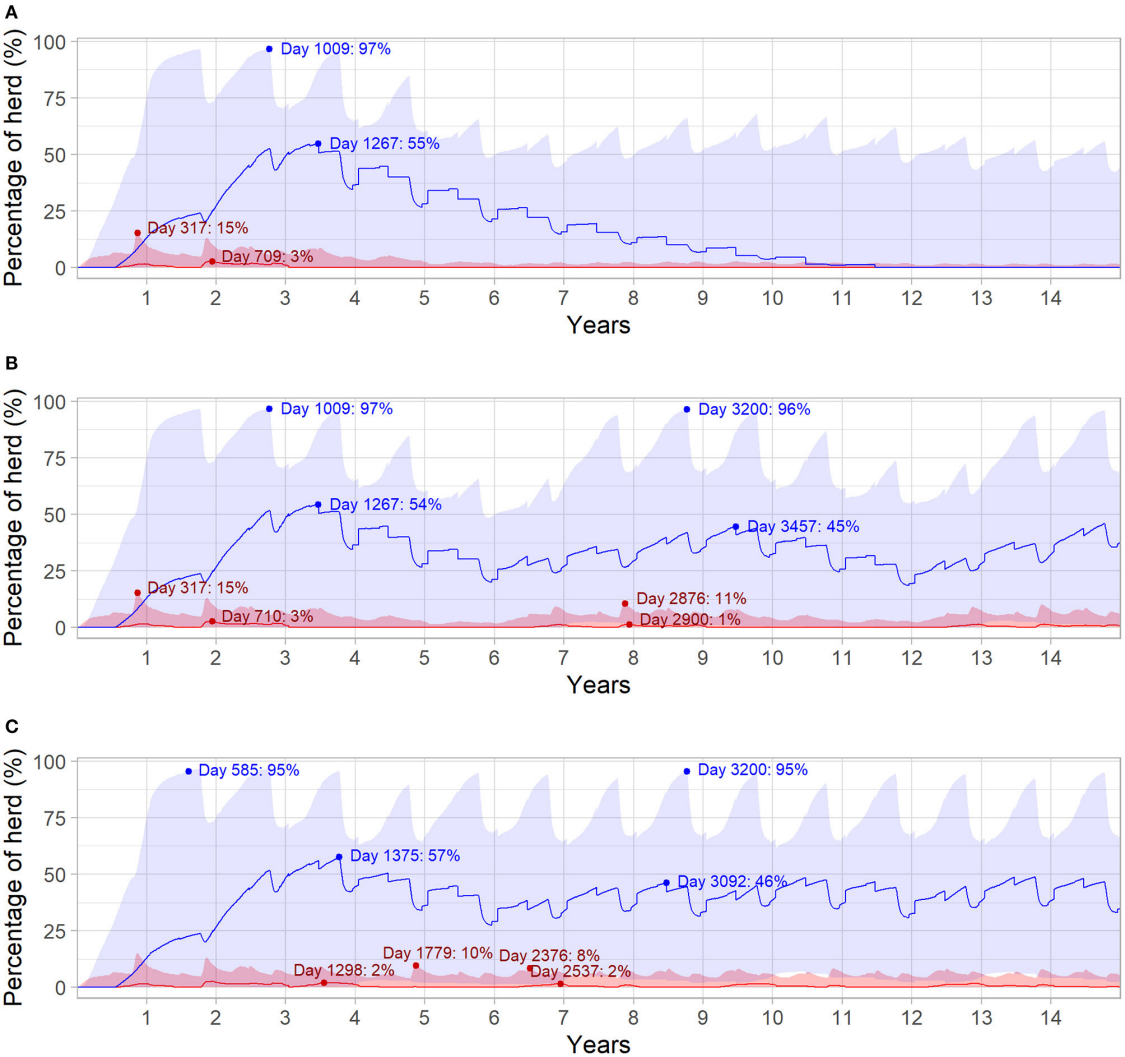
Daily infection dynamics for different BVDV introduction scenarios over 15 years in a simulated beef herd. Introduction of a single PI animal in the first year of the simulation (Scenario 1; **A**), introduction of a single PI animal every 6 years (Scenario 2; **B**) and introduction of a single PI animal every 3 years (Scenario 3; **C**) for 15 years. Percentage of daily viremic (TI and PI) animals (red) and daily seropositive animals (blue), with maternal immunity not represented. Solid red and blue lines indicate the median value for viremic and seropositive animals, respectively, while shaded areas represent the 95% range. Dot points and annotations represent the highest median values following each introduction of a PI animal (values following initial PI introduction in Scenario 3 are removed for improved readability).

Figure 4 highlights the relationship between herd immunity and the prevalence of viremia for the different outbreak scenarios. In all three scenarios, initial introduction of BVDV in the naïve herd resulted in maximum daily prevalence of viremic animals at median 3% (95% range 0.30–15%) of the total herd. Herd seroprevalence was highest at the start of the third year of the simulation (median 55%, 95% range 0.60–97%), following a steady decline in immunity as animals left the herd. In Figures 4B,C, BVDV was introduced in 6- and 3-year increments after the first incursion, respectively. In both scenarios, the second incursion resulted in a smaller increase in viremic animals (median 1%, 95% range 0.30–11% and median 2%, 95% range 0.60–10% for six and three years, respectively) compared to the initial introduction. The highest daily prevalence of viremia was even lower for subsequent BVDV introductions every three years (median 2%; 95% range 0.40–8%; Figure 4C).

### BVDV Influence on Production Outputs

**Figure 5:**
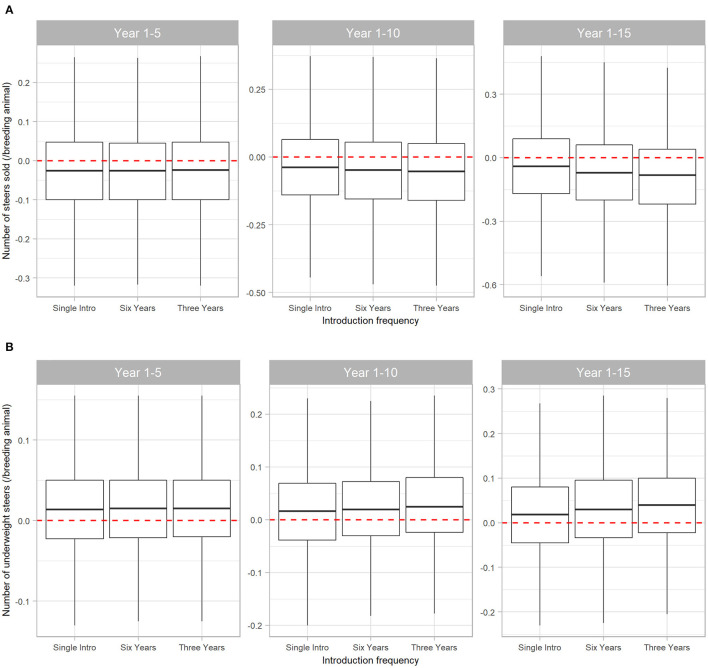
Boxplot illustrating the cumulative effect of BVDV introduction frequency on production outputs relating to steer sales in a simulated beef herd. The values depict the production loss for BVDV introduction compared to a disease-free herd. The dotted red line highlights zero, which would represent “no difference” between the disease-free scenario and the scenarios with BVDV. Values either side of the line indicate the impact of BVDV on the number of steers sold **(A)** and the number of steers sold that were too lightweight to qualify for a price premium **(B)**/breeding animal. Boxes represent the 25 and 75% percentiles while whiskers represent the 2.5 and 97.5% percentiles. Outliers removed for improved scale and readability.

The number and weight of steers sold were most affected by BVDV. Initial introduction of BVDV in a naïve herd resulted in a reduction of steers sold at median 0.025 steers (95% range −0.32 to 0.270) per breeder over five years. Of these steers, median 0.015 steers/breeding animal (95% range −0.130 to 0.155) had liveweight at sale insufficient to reach the premium price range. Figure 5 demonstrates that the negative effects of BVDV on steer sales are cumulative as introduction of the disease becomes more frequent, with a reduction of median 0.082 (95% range −0.605 to 0.425) steers sold/breeding animals in Scenario 3 over 15 years (this equates to 4 and 66 steers for a 50- and 800-breeder herd, respectively).

**Figure 6:**
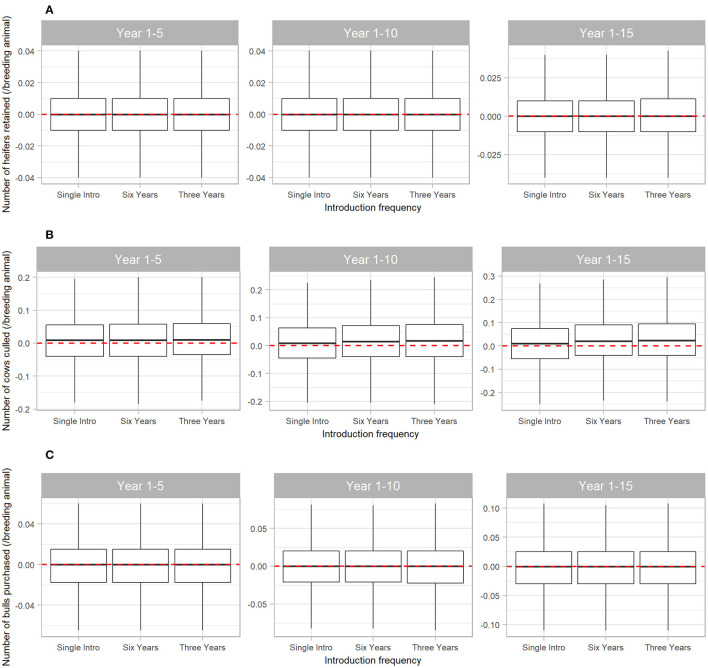
Boxplot illustrating the cumulative effect of BVDV introduction frequency on economic outputs relating to reproduction animals in a simulated beef herd. The values depict the production loss in for BVDV introduction compared to a disease-free herd. The dotted red line highlights zero, which would represent “no difference” between the disease-free scenario and the scenarios with BVDV. Values either side of the line indicate the impact of BVDV on the number of heifers retained for breeding **(A)**, the number of cows culled **(B)** and the number of bulls purchased for replacement **(C)**/breeding animal. Boxes represent the 25 and 75% percentiles while whiskers represent the 2.5 and 97.5% percentiles. Outliers removed for improved scale and readability.

The number of cows culled from the herd was also affected by BVDV, but to a lesser extent than steer sales (Figure 6B). Compared to a disease-free herd, BVDV resulted in median 0.01 (95% range −0.18 to 0.20) more cows culled/breeding animal in the first five years of disease exposure (all Scenarios) and median 0.024 (95% range −0.24 to 0.30) more cows culled/breeding animal when BVDV was introduced every three years for 15 years (Scenario 3). BVDV presence and introduction frequency had no influence on the median number of heifers retained in the herd or bulls purchased over 5, 10 or 15 years (Figures 6A,C).

### External Validation of Model Outputs

A description of the model outputs and reference values used for external validation of the model are summarized in Table 5. The mean herd viremic prevalence (1%), median age of PI animal death (6.7 months) and the median herd seroprevalence two years after a single BVDV introduction (40%) were all analogous to the corresponding median/mean values obtained in the literature. The mean prevalence of PI animals (0.7%), the median pregnancy risk (88%) for a naïve herd and the calf mortality rate in an infected herd (5%) was also within the range of the external reference values (0.2–0.8%; 85–89% and 5–7%, respectively) (64, 65). The median value for abortion rate, calf mortality rate and the percentage of light sale steers following a single BVDV introduction did not match the external data; however, the 95% range of these parameters did include their corresponding reference value.

**Table 5:** Comparison of model outputs to corresponding values from the published literature for external validation of a within-herd BVDV simulation model. All model outputs are presented as median (95% range) unless otherwise indicated.

**Parameter**	**Simulation model**		**Reference**		
	**Description**	**Value**	**Value**	**Description**	**Source**
Viremia	Prevalence of viremic animals in an endemic setting (Scenario 3) for 15-year period	1%^*^ (0–7)	1%^*^ (0–5)	Reported prevalence of viremic animals in Australia	(64)
	All Introduction Times and herd sizes				
	Self-elimination (years) of BVDV following a single introduction (Scenario 1)	4 **(1–15**^a^**)**	**6–8**	The time (years) between BVDV Ab detection and absence of Ag-positive animals in a NSW 400-breeder beef herd with no BVDV control	(65)
	Introduction Time 5 and 400-breeder herd				
Seroprevalence	Prevalence of seropositive animals in an endemic setting (Scenario 3) for a 15-year period	37%^*^ **(0–86)**	53%^*^ (43–62)	Reported prevalence of Ab positive animals in Australia	(64)
	All Introduction Times and herd sizes				
	Prevalence of seropositive animals the second year after a single introduction (Scenario 1)	**40%** (0–96)	**42%**	Herd seroprevalence two years after suspected time of infection in a breeding herd of 400 animals	(65)
	Introduction Time 5 and 400-breeder herd				
	Prevalence of seropositive animals in the fourth year after a single introduction (Scenario 1)	52% **(0–78)**	**75%**	Herd seroprevalence four years after suspected time of infection in a breeding herd of 400 animals	(65)
	Introduction Time 5 and 400-breeder herd				
PI animals	Prevalence of PI animals in an endemic setting (Scenario 3) for a 15-year period using all herd sizes and introduction times	0.7%^*^ (0.0–3.8)	0.5%^*^ **(0.2–0.8)**	Reported prevalence of PI animals in Australia	(64)
			**0.9%** (0.0–3.0)	Prevalence of PI calves supplied to the Tick Fever Research Centre QLD from 1990 to 1996	(66)
	Age of death (months) for PI animals	**6.7** (0.4–18.9)	**6.5**	The age (months) at which half of all PI animals are expected to die in 10 danish herds	(50)
	All Scenarios, Introduction Times and herd sizes				
Pregnancy risk	Annual pregnancy risk with no active BVDV infection (Scenario 0).	**88%** (84–91%)	**85–89%**	Pregnancy risk for breeding herd in NSW beef herd of 400-breeders	(65)
	No Introduction Time and 400-breeder herd				
	Decrease in pregnancy risk due to a single BVDV introduction (Scenario 1)	1% (−3 to 4)	2–7%	Difference in pregnancy risk following the suspected time of BVDV exposure in a QLD herd with 800-breeders^b^	(52)
	Introduction Time 2 and 800-breeder herd				
Abortion rate	Increase in abortion as a result of a single BVDV introduction (Scenario 1).	1% **(−2 to 6)**	**5%**	The foetal losses attributed to BVDV exposure between day 51 and 210 of gestation for 207-breeders in QLD	(67)
	Introduction Time 1 and 200-breeder herd				
Calf mortality rate	Annual calf mortality with no active BVDV infection (Scenario 0)	3% **(1.2–5)**	**1–2%**	Normal calf mortality rate in a NSW beef herd of 400-breeders	(65)
	No Introduction Time and 400-breeder herd				
	Increase in calf mortality due to a single BVDV introduction (Scenario 1).	**5%** (2–8)	**5–7%**	Calf mortality rate expected to be due to BVDV following an outbreak in a NSW beef herd of 400-breeders	(65)
	Introduction Time 1 and 400-breeder herd				
Marking rate	Decrease in branding rate due to a single BVDV introduction (Scenario 1)	2% **(−4 to 9)**	**3%**	Difference in branding rate for unvaccinated (against BVDV) heifers to vaccinated heifers in a study examining vaccine efficacy in Australia	(68)
	Introduction Time 1 and 800-breeder herd				
			**4%**	Decrease in branding rate suspected to be due to a BVDV outbreak in a QLD herd with 800-breeders	(52)
Sale animals	Percentage of lightweight steers at sale following a single BVDV introduction (Scenario 1)	3% **(−4 to 10)**	**6%**	Percentage of steers estimated to be lightweight at slaughter (at 2–3 years old) following an outbreak of BVDV in a QLD beef herd with 900-breeders	(52)
	Introduction Time 5 and 800-breeder herd				

*Bold = Analogous model and reference values*.

* *Mean value used instead of median*.

a *Virus elimination did not occur during 15-year simulation*.

b *Exact figures were not available for pre-BVDV exposure in this case study*.

ab *antibody; Ag, antigen, QLD, Queensland, Australia; NSW, New South Wales, Australia*.

### Sensitivity Analysis

Sensitivity analysis was conducted using Scenario 1 (most variation in model outputs). Scatter plots indicated no evidence of collinearity and so all parameters in Supplementary Table 4 were included in the analysis. The influence of the input parameters on model outputs of interest are illustrated in Supplementary Figures 5–10. Introduction time of the virus, breeding herd size and the number of effective contacts for PI animals resulted in the most variation in the total number of animals infected over 15 years. The adult mortality rate and the calf mortality rate resulted in the greatest variation for the total number of steers sold, and annual calf mortality was also the biggest contributor to the total number of underweight steers sold. The number of heifers retained for breeding and the number of cows culled was most dependent on breeding herd size and the mortality rate of adult cattle. Apart from breeding herd size, all other input variables had a negligible impact on the total number of bulls purchased in the simulation period.

## Discussion

The individual producer is solely responsible for the prevention and control of BVDV in Australia. This novel disease simulation model reflects the impact of BVDV in settings consistent with Australian beef production at the individual farm level. Convergence testing demonstrated stability in all of the model outputs, illustrating little variation between simulations. Model outputs indicated that a single BVDV incursion is likely to cause the birth of at least one PI animal (meaning that infection is established in the herd) in ~60% of all simulations. The timing of BVDV introduction in relation to the production calendar had the most influence on establishment, which was most likely for the 73 days prior to joining through to 146 days after the joining period. This is recognized as the highest risk period for persistent infection in developing fetuses (8, 69, 70). Introduction of animals is one of the main pathways in which BVDV can enter a herd and it is common for Australian beef producers to purchase breeding animals (such as bulls or replacement heifers) in conjunction with the joining period (27). This model demonstrates that an established BVDV outbreak is likely to occur in a beef farm with no preventative measures in place, especially when replacement practices coincide with joining.

Once BVDV has become established in a beef herd, the model demonstrates that herd size characterizes the severity and length of the outbreak. The proportion of animals in the simulated farm that were viremic at the peak of infection had an inverse relationship to breeding herd size; however, the distribution of MCs in simulated paddocks may have contributed to these results. The number of MCs in each paddock is fixed in this model, based on the bull-to-cow ratio. Smaller herds will have a higher proportion of breeding animals in a single paddock when compared to a large herd, in which the breeding herd is spread across multiple paddocks. BVDV transmission within a paddock will initially result in a higher proportion of infected animals in a smaller herd. Transmission within a paddock diminishes over time as animals become immune to infection and so, transmission between paddocks becomes responsible for BVDV persistence within the farm. Therefore, the duration of a BVDV outbreak in the model increased as herd size increased. A herd of 50-breeders had a maximum outbreak duration of 8 years following a single introduction, compared to an 800-breeder herd, in which 23% of outbreaks continued after 15 years. In reality, it is reasonable to assume that larger farms would consist of more paddocks than those with smaller herds to achieve ideal stocking rates to maximize production (71). The relationship between herd size, outbreak severity and virus persistence demonstrated by the results of this study suggest that timely elimination of BVDV may require less intervention in smaller herds.

While the median duration of BVDV outbreak in the simulated 400-breeder herd (4 years) was shorter than that reported for a similar sized Australian outbreak, the 95% range included the published values for elimination time [6–8 years; Allworth et al. (65)]. In the case study, Allworth et al. (65) speculates that BVDV was introduced into the NSW farm *via* breeding stock. It was not possible to ascertain the infection status of these animals retrospectively, and it is possible that introduction of multiple infected animals may have contributed to a longer time to elimination. In addition to elimination time, data from the model for the 400-breeder herd showed that the 2-and 4-year seroprevalence in the 73-day period prior to joining were comparable to the values reported by Allworth et al. (65). The model also demonstrated that the highest daily prevalence of viremia following subsequent BVDV introductions became lower as herd seroprevalence increases; a relationship which is well recognized in the literature (11). These examples, as well as those found in Table 5, further validate the disease transmission component of the BVDV simulation model described in this study.

Dairy, intensive beef and extensive beef farming operations require different approaches to BVDV detection and prevention as a result of variations in farm management (20, 21, 69, 72). Due to the subclinical nature of BVDV, in most cases interventions on an individual farming level are not considered until an effect on production is identified (73–75). In Australia there is minimal contact between beef producers and cattle between management events and so producers are unlikely to recognize a subtle drop in annual production (76). The annual impact of BVDV on pregnancy loss, abortion and calf mortality recorded by the simulation model was modest, even at its highest following initial exposure of a naïve herd. However, the study demonstrated that the impact of repeat BVDV incursions as little as every three years can result in long-term impacts that would affect beef farm productivity; in particular the number of steers sold and the liveweight of those steers. Economic analysis is needed to determine whether these long-term production impacts reduce farm revenue enough to prompt action by the producer.

Long et al. (77) found that BVDV is of low concern to 68% of Australian beef producers, with other diseases and management issues taking priority. Motivating producers to address BVDV is a challenge that is not specific to Australia (75, 78). Apart from undetected losses, difficulty in communicating the negative impacts of BVDV to stakeholders is partly due to the wide variation in outcomes that could occur during an outbreak of BVDV. Poor conception and reduced pregnancy risks are often identified during BVDV outbreaks (70); however, this study, consistent with Allworth et al. (65), found that the pregnancy risk in a herd of 400 animals was largely unaffected by BVDV. The joining practices used in this model and described by Allworth et al. (65) are similar to industry-recommended joining practices for seasonal calving beef herds in NSW (McConchie 2007, MLA 2019). These short duration joining periods are designed to limit infertility and might also reduce the apparent reproductive effects of BVDV infection. If pregnancy risks are unaffected, this could explain the marginal impact of simulated BVDV infection on culling rates, which are largely dictated by pregnancy status (46). Given the aforementioned relationship between the timing of reproductive practices and disease behavior, any discrepancies between the reproductive results of this study and the published effects of BVDV could be due to differences in reproductive management and/or model uncertainty; both of which should be considered when interpreting model outcomes. However, the model has identified a range of possible consequences of BVDV infection that might be used as best- and worst-case scenarios to inform decision-making for seasonal-calving Australian beef farms.

A limitation of this model is an inability to account for the impact of secondary infections that may exacerbate the effects of BVDV. Immunosuppression is a documented consequence of BVDV infection that is suggested to facilitate secondary infections that may increase abortion, calf mortality and reduce growth performance (79–81). In this study, immunosuppression only affects growth rate and therefore, it is likely that the model underestimates the impact that BVDV might have on productivity in the field. Another limitation of the model is the use of a NZ parameter for BVDV transmission. Whilst this is based on extensive production, it should be noted that this model does not account for the effect of the Australian climate on virus survival. Furthermore, a limitation of individual-based models is the assumption of homogenous mixing between animals within a group. However, in reality, beef cattle exhibit a social hierarchy which results in clustering of animals within a group which might affect disease transmission (82). Future studies modeling BVDV in Australia would benefit from serological data which could be used to identify transmission characteristics of the disease specific to the Australian environment.

Using simulation modeling, this study described the impact of BVDV on a farm characteristic of Australian beef farming. Exposure to BVDV is most likely to cause an outbreak when the virus is introduced in conjunction with the breeding period and there is a high (60%) likelihood that BVDV will be established in a beef herd after the introduction of a single PI animal. The initial impact of a BVDV outbreak will be more severe in smaller beef herds; however, the virus takes longer to self-eliminate as herd size increases. The model can measure the underlying production losses that can occur from long-term outbreaks of BVDV which might otherwise go unnoticed in the field; however, economic analysis is required to determine the financial implications of BVDV in Australian beef production. Future studies can use this model to investigate BVDV prevention strategies for Australian beef farms.

## Data Availability Statement

The raw data supporting the conclusions of this article will be made available by the authors, without undue reservation.

## Author Contributions

The study was designed by JF, VB, MH-J, JM and YM as part of a PhD project. Modeling and data analysis were performed by JF, with assistance from VB, CK, and TH. All authors read and approved the final manuscript.

## Funding

This study is part of a PhD project that was supported by an Australian Research Training Program (ARTP) scholarship, with a top-up from the Graham Centre for Agricultural Innovation and Meat and Livestock Australia Ltd. There was no involvement of the funding sources in the study design, collection or analysis of data, interpretation of results, or the decision to submit the article for publication.

## Conflict of Interest

The authors declare that the research was conducted in the absence of any commercial or financial relationships that could be construed as a potential conflict of interest.

## Publisher's Note

All claims expressed in this article are solely those of the authors and do not necessarily represent those of their affiliated organizations, or those of the publisher, the editors and the reviewers. Any product that may be evaluated in this article, or claim that may be made by its manufacturer, is not guaranteed or endorsed by the publisher.

## Abbreviations

BVDV: bovine viral diarrhea virus
PI: persistently infected
TI: transiently infected
NSW: New South Wales
B: bulls
CC: cull cows
WS: weaned steers
WH: weaned heifers
FC: first-calving cows
MC: mature cows
CF: calf at foot
BRD: bovine respiratory disease
GAM: generalized additive mixed
CV: coefficient of variation
NZ: New Zealand.
