# Supplementary material for: Modeling the Effect of Bovine Viral Diarrhea Virus in Australian Beef Herds
Source: Front Vet Sci. 2021 Dec 14;8:795575. doi: 10.3389/fvets.2021.795575 (PMC8712561; doi:10.3389/fvets.2021.795575)
Supplement: Supplementary file 1 [file Data_Sheet_1.pdf]

Supplementary Material

1 Supplementary Figures and Tables

1.1 Supplementary Tables

Supplementary Table 1: Proportional calf outcomes due to foetal infection with BVDV by month of gestation.

	Month of gestation								
**Calf outcome**	**1**	**2**	**3**	**4**	**5**	**6**	**7**	**8**	**9**
Aborted	0.20	0.15	0.15	0.15	0.15	0.05	0.05	0.00	0.00
PI	0.20	0.85	0.60	0.25	0.00	0.00	0.00	0.00	0.00
Weak	0.05	0.00	0.25	0.55	0.30	0.20	0.00	0.00	0.00
Immune	0.55	0.00	0.00	0.05	0.55	0.75	0.95	1.00	1.00

Supplementary Table 2: Weight parameters of healthy and BVDV infected cattle based on industry standards for Australian beef cattle used to calculate daily weight gain percentages in Supplementary Table 3.

**Parameter**		**Targets (kg)**
Average calf birthweight	Healthy	PERT (32, 36, 40)
220-day average live weight	Steers (cow-reared)	231
	Heifers (cow-reared)	215
	Morbid steers (cow-reared)	174
	Morbid heifers (cow-reared)	162
	Steers (heifer-reared)	204
	Heifers (heifer-reared)	191
	Morbid steers (heifer-reared)	153
	Morbid heifers (heifer reared)	143
Heifer weight at puberty		270
450-day average live weight	Steers	425
	Heifers	290
	Morbid steers	280
	Morbid heifers	190
730-day live weight	Heifers	U (420, 450)
Mature weight	Cows	U (460, 520)
	Bulls	850

Supplementary Table 3: Animal-specific proportion of individual animal liveweight for daily weight gain, used to model BVDV in an Australian beef herd.

Age		Value	References
Birth to 220 days	Steers (cow-reared)	8.48×10^{-3}	Author derived
	Heifers (cow-reared)	8.16×10^{-3}	
	Morbid steers (cow-reared)	7.19×10^{-3}	
	Morbid heifers (cow-reared)	6.86×10^{-3}	
	Steers (heifer-reared)	7.92×10^{-3}	
	Heifers (heifer-reared)	7.62×10^{-3}	
	Morbid steers (heifer-reared)	6.60×10^{-3}	
	Morbid heifers (heifer reared)	6.29×10^{-3}	
220 days to 450 days	Steers	2.66×10^{-3}	
	Heifers	1.30×10^{-3}	
	Morbid steers	2.08×10^{-3}	
	Morbid heifers	7.00×10^{-4}	
450 days to 730 days	Heifers	5.40×10^{-4}	
730 days to max weight	Cows	4.60×10^{-4}	

Description of liveweight gain proportions obtained from target weights.

Liveweight gain proportions were calculated separately for each animal type to account for the variation in growth rate that occurs between different life stages, that would not be possible if using a uniform proportion for all age groups. The use of different proportions to calculate daily weight gain also allows for a reduced proportion to simulate reduced growth rates due to morbidity/immunosuppression, which may occur through infection with secondary disease.

The proportion of daily liveweight gain for each animal type (healthy and infected) was calculated using the target bodyweights in Supplementary Table 2. To calculate these proportions, 1000 animals were created in R with a liveweight between 32 and 40kg (PERT distribution as per the gestation length for a cow).The weight of each animal was then multiplied by number in a grid containing all values between 0 and 1 and the result was added to the individual animal weight. This was repeated for 220 days until the average 220-day liveweight (and range of expected liveweights) was recorded for each value in the grid. The value that resulted in the closest liveweight to the target 200-day liveweight was used as the animal-specific proportion for daily liveweight gain in the model.

This was repeated for intervals: 220 to 450-day liveweight, 450 to 730-day liveweight and 730-day to mature weight for healthy vs infected and cow-reared vs heifer-reared calves.

Supplementary Table 4: A description of the inputs used in the sensitivity analysis for a BVDV simulation model.

**Model inputs**	**Description**	**Distribution**
Breeding herd size	The size of the breeding herd.	Discrete (50, 100, 200, 400, 800)
Introduction time	The time of year that the disease was introduced.	Discrete (1, 2, 3, 4, 5)
PI (within) effective contacts	The number of effective contacts for PI animals within a group.	PERT (0.03, 0.11, 0.50)
Infectious period	The number of days that a TI animal will shed the virus.	PERT (7, 10, 14)
Maternal antibody duration	The number of days that a calf is immune due to maternal antibodies.	PERT (120, 180, 240)
Morbidity rate (Adult)	The percentage of TI mature cattle that will be immunosuppressed when infected.	Uniform (0.10, 0.30)
Morbidity rate (Calf)	The percentage of TI calves that will be immunosuppressed when infected.	PERT (0.02, 0.24, 0.69)
Mortality rate (Adult)	The annual mortality rate of adult cattle.	PERT (0.008, 0.017, 0.024)
Mortality rate (Calf)	The annual mortality rate of healthy calves.	PERT (0.030, 0.045, 0.060)
Mortality rate (Morbid)	The annual mortality rate of morbid cattle.	PERT (0.35, 0.50, 0.66)
Infertile period	The number of days that a TI female will have a reduced conception rate.	Uniform (42, 60)

1.2 Supplementary Figures

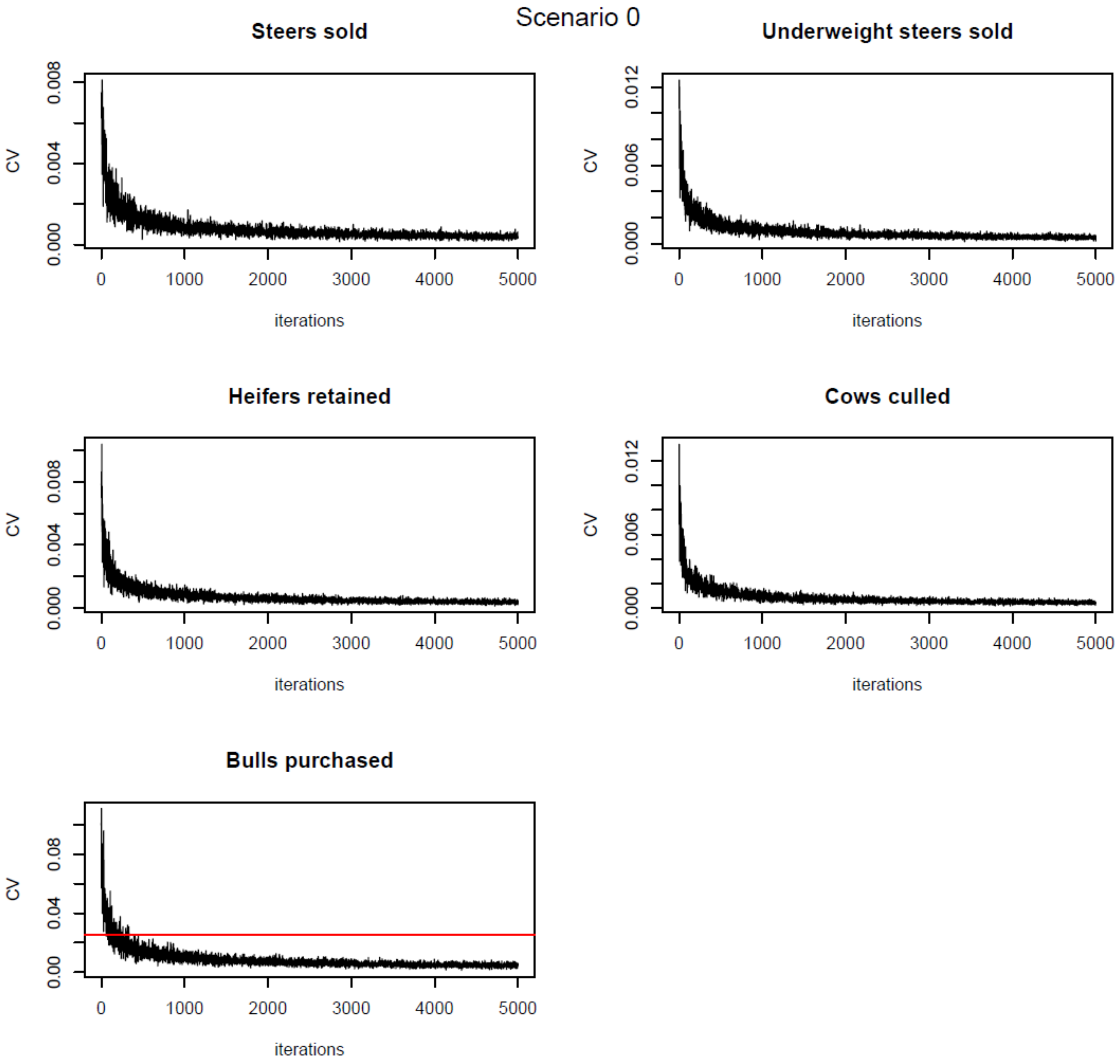

Supplementary Figure 1. Frequency plot illustrating convergence of the coefficient of variation (CV) for the model outputs of interest for Scenario 0. The number of iterations was deemed sufficient when the CV was <0.025 (indicated by the horizontal red line on each plot).

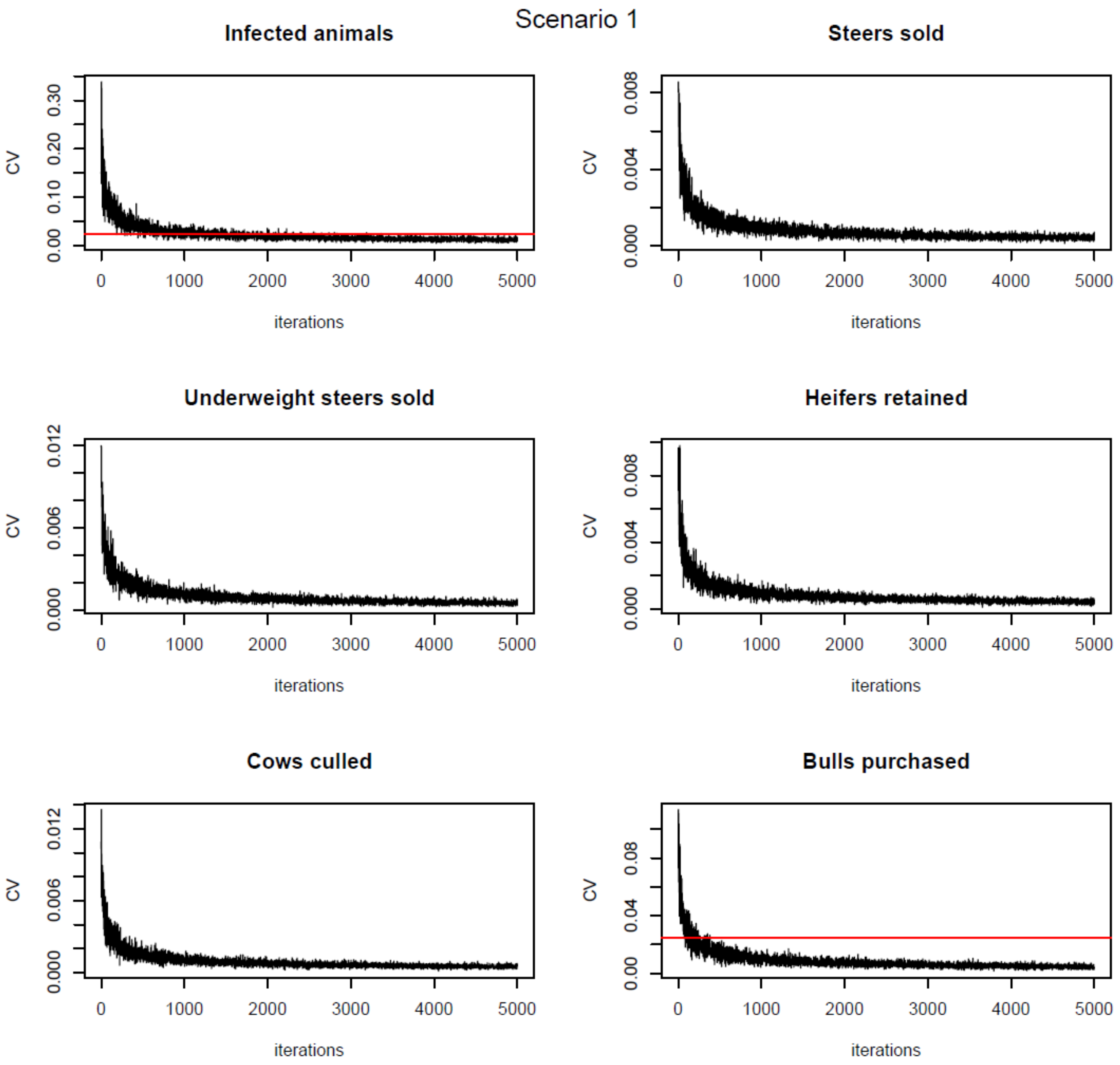

Supplementary Figure 2. Frequency plot illustrating convergence of the coefficient of variation (CV) for the model outputs of interest for Scenario 1. The number of iterations was deemed sufficient when the CV was <0.025 (indicated by the horizontal red line on each plot).

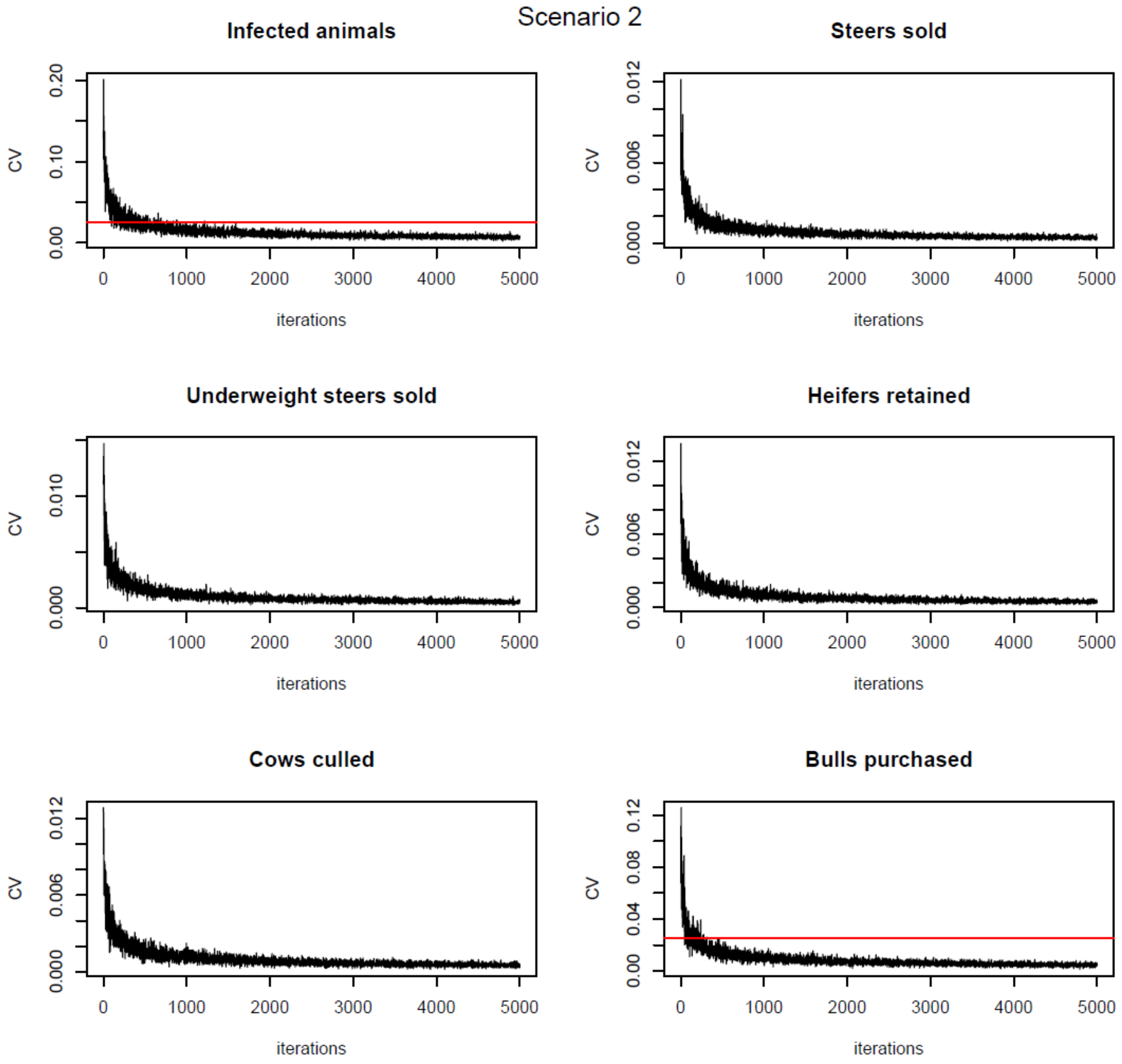

Supplementary Figure 3. Frequency plot illustrating convergence of the coefficient of variation (CV) for the model outputs of interest for Scenario 2. The number of iterations was deemed sufficient when the CV was <0.025 (indicated by the horizontal red line on each plot).

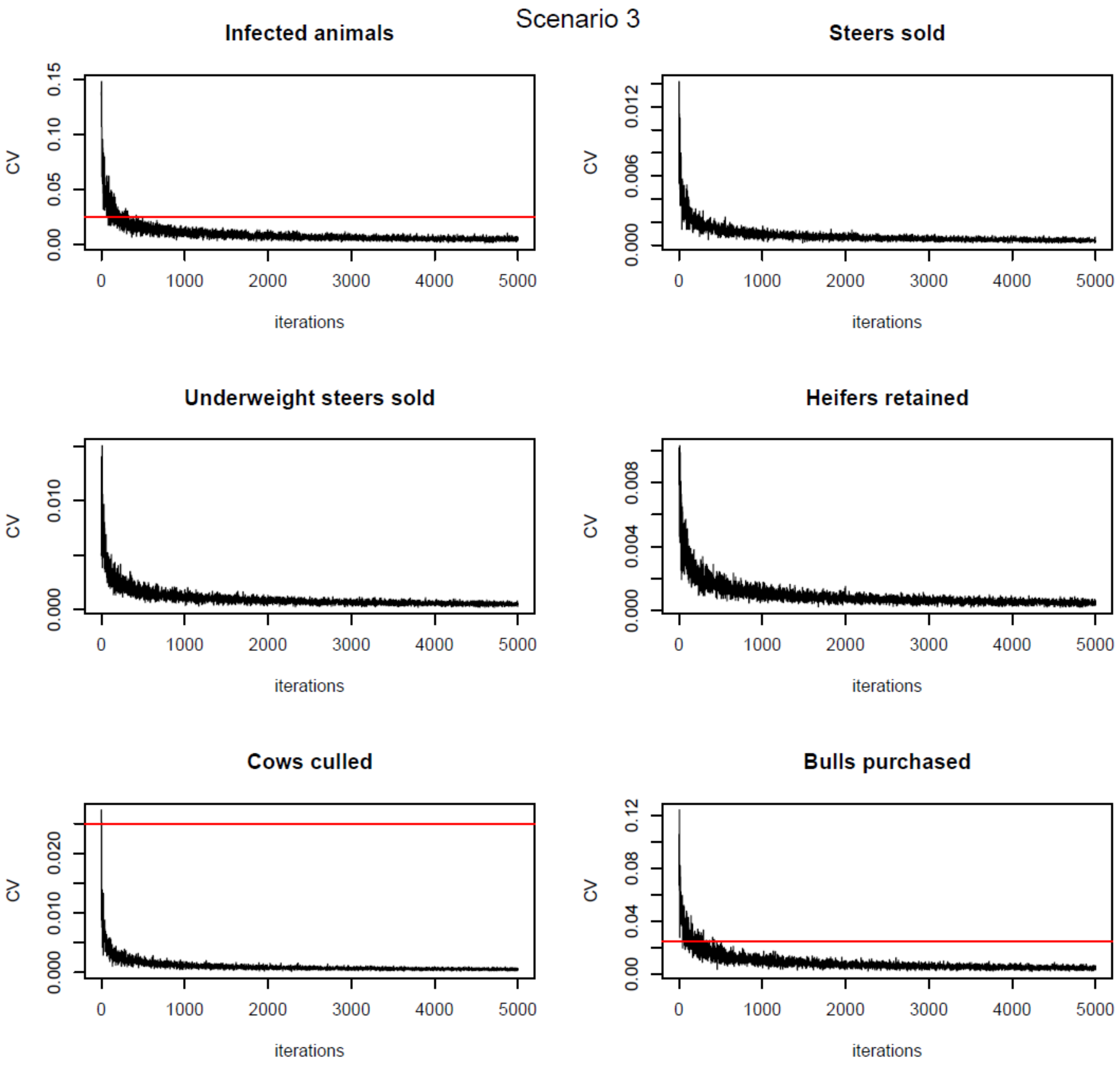

Supplementary Figure 4. Frequency plot illustrating convergence of the coefficient of variation (CV) for the model outputs of interest for Scenario 3. The number of iterations was deemed sufficient when the CV was <0.025 (indicated by the horizontal red line on each plot).

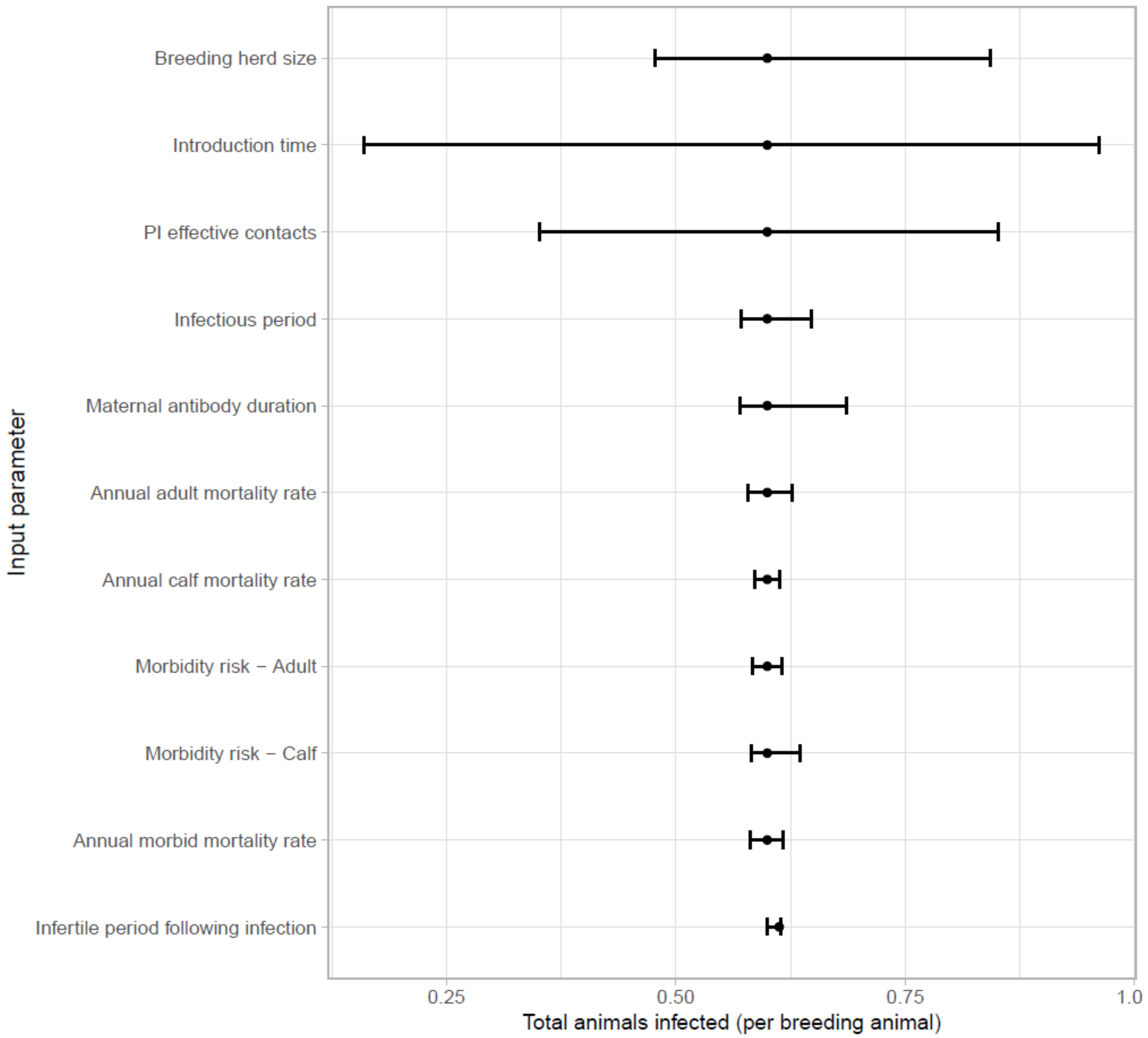

Supplementary Figure 5. Error bar plot illustrating the contribution of model inputs on the total number of infected animals after 15 years following a single PI introduction (Scenario 1).

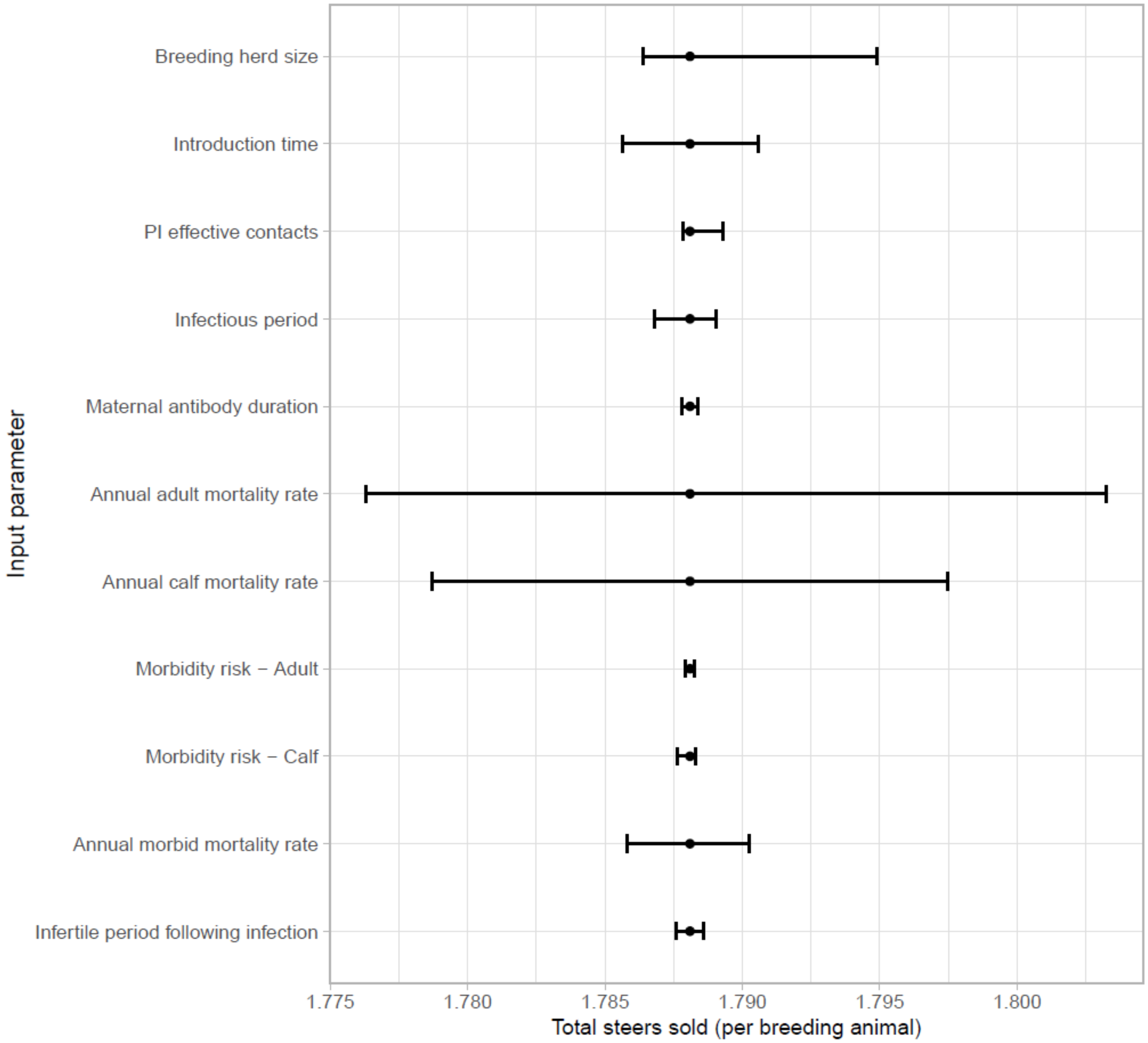

Supplementary Figure 6. Error bar plot illustrating the contribution of model inputs on the total number of steers sold over 15 years following a single PI introduction (Scenario 1).

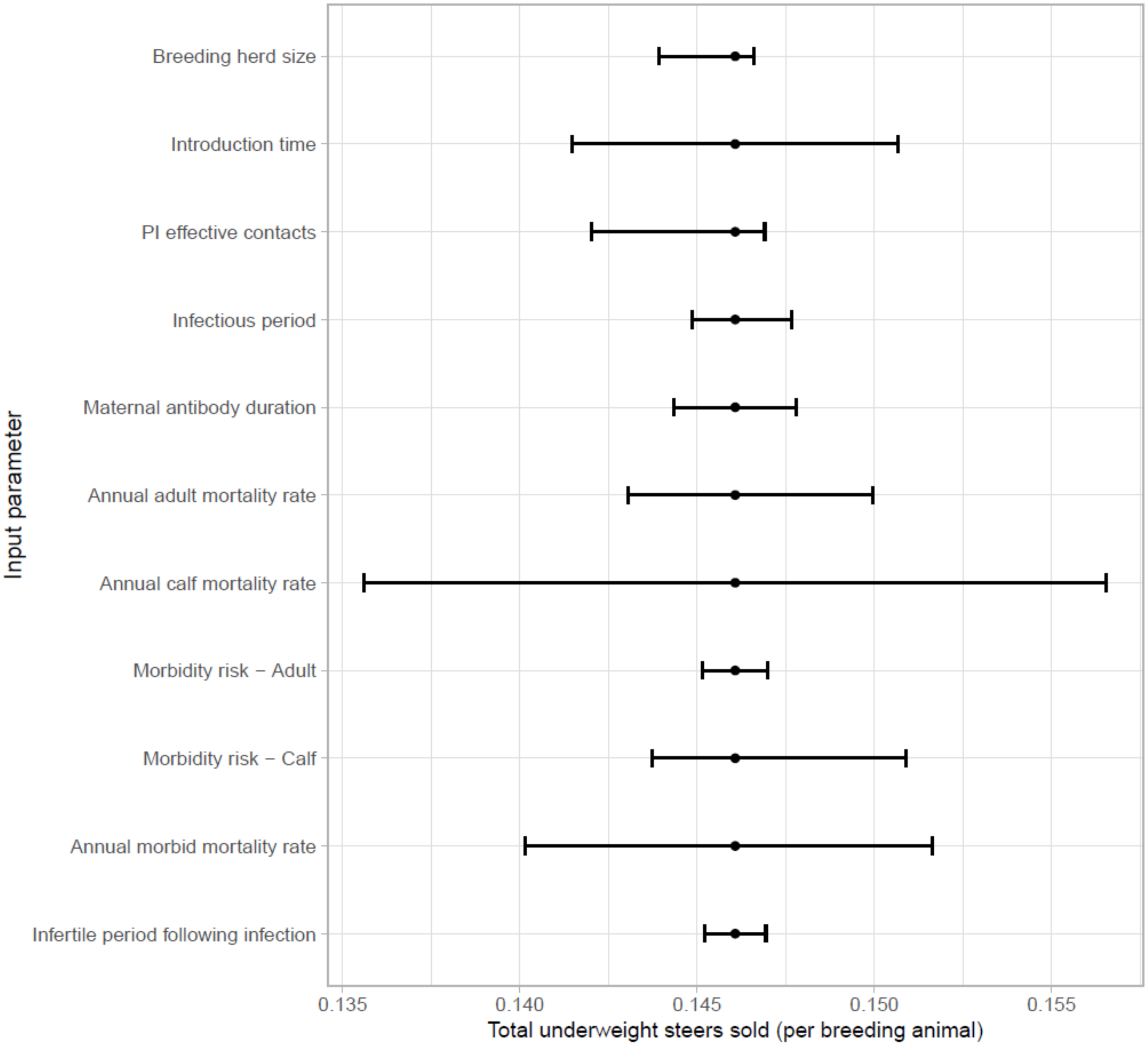

Supplementary Figure 7. Error bar plot illustrating the contribution of model inputs on the total number of underweight steers sold over 15 years following a single PI introduction (Scenario 1).

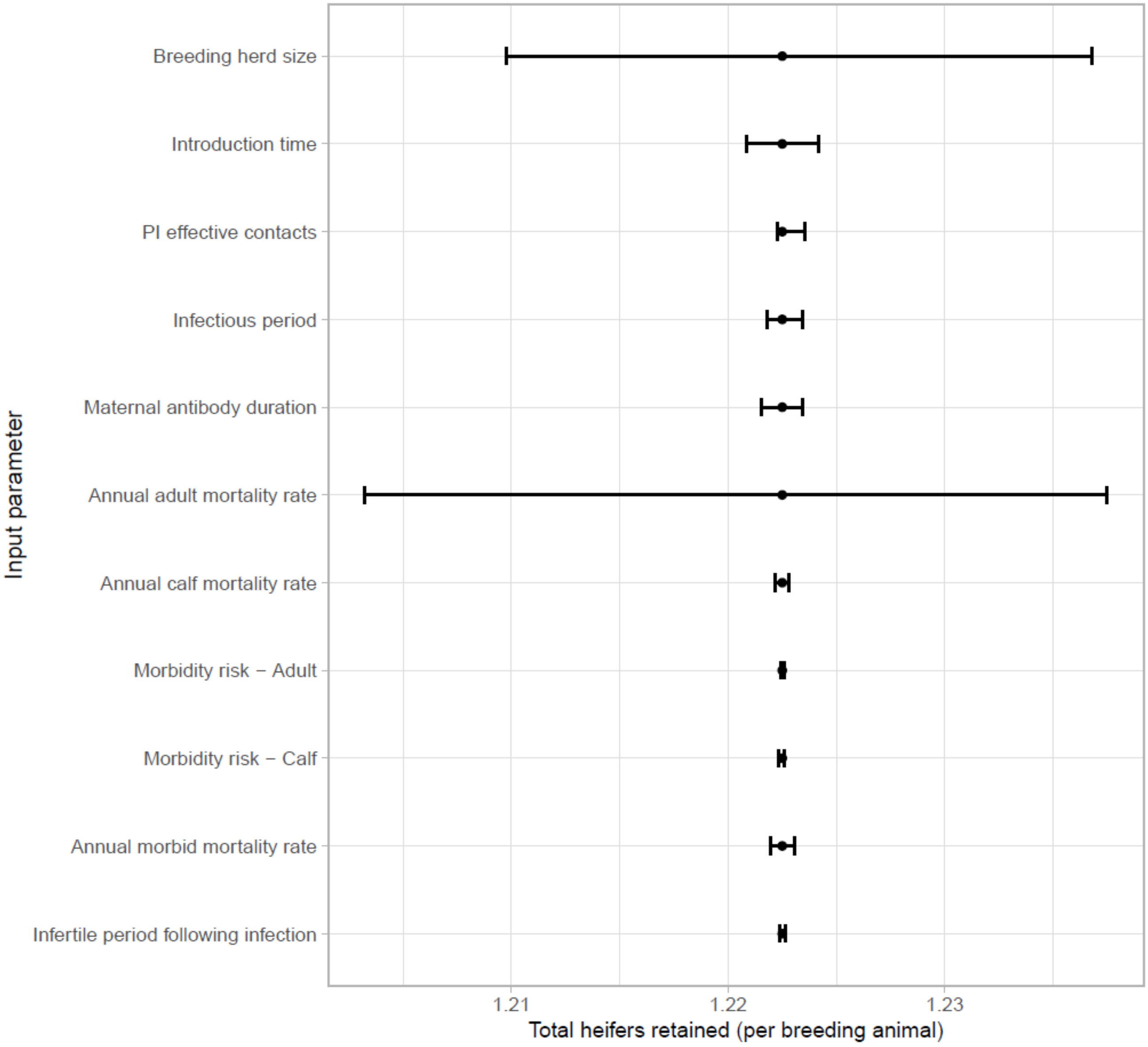

Supplementary Figure 8. Error bar plot illustrating the contribution of model inputs on the total number of heifers retained over 15 years following a single PI introduction (Scenario 1).

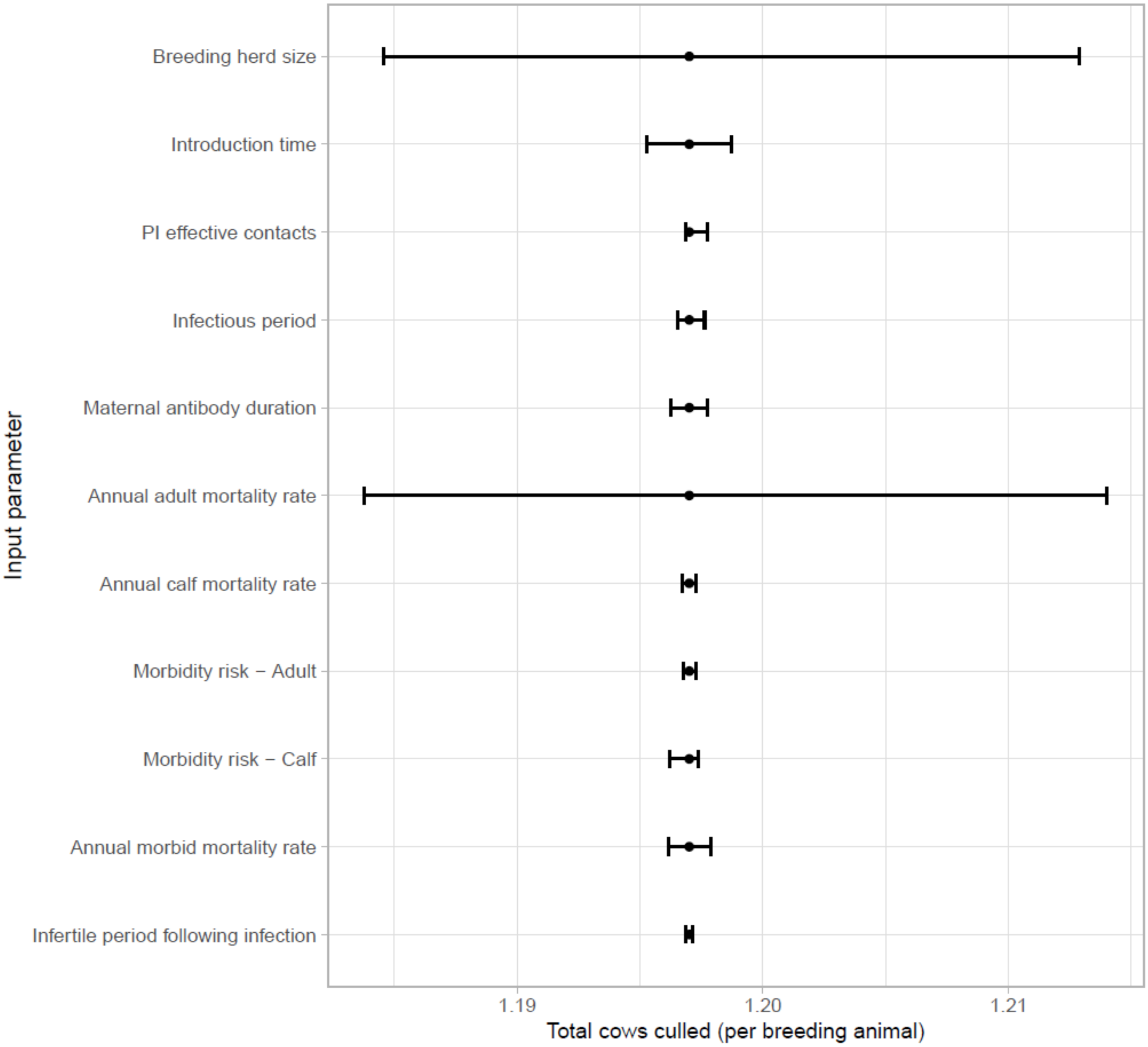

Supplementary Figure 9. Error bar plot illustrating the contribution of model inputs on the total number of cows culled over 15 years following a single PI introduction (Scenario 1).

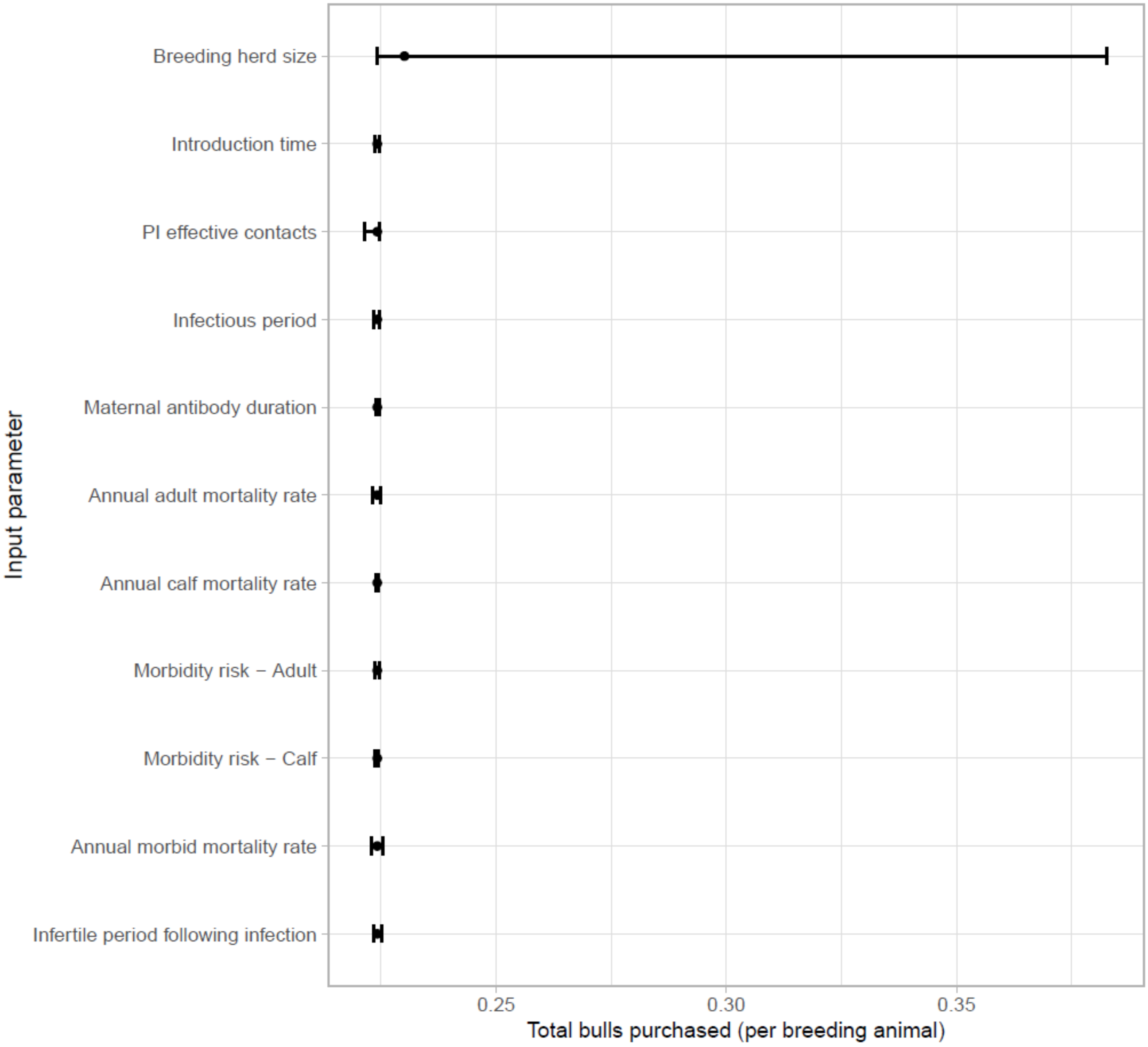

Supplementary Figure 10. Error bar plot illustrating the contribution of model inputs on the total number of bulls purchased over 15 years following a single PI introduction (Scenario 1).
